# Substrate recognition by human separase

**DOI:** 10.1126/sciadv.ady9807

**Published:** 2025-11-12

**Authors:** Jun Yu, Sophia Schmidt, Margherita Botto, Kitaik Lee, Chloe M. Ghent, Jonah M. Goodfried, Andrew Howe, Francis J. O’Reilly, David O. Morgan, Andreas Boland

**Affiliations:** ^1^ Department of Molecular and Cellular Biology, University of Geneva, Geneva, Switzerland.; ^2^ Center for Structural Biology, Center for Cancer Research, National Cancer Institute (NCI), Frederick, MD 21702-1201, USA.; ^3^ Department of Physiology, University of California, San Francisco, San Francisco, CA 94143, USA.; ^4^ Bioimaging Center, University of Geneva, Geneva, Switzerland.

## Abstract

The cohesin complex encircles sister chromatids in early mitosis. At anaphase onset, sister separation is triggered by the proteolytic cleavage of the cohesin subunit SCC1/RAD21 by separase. SCC1 contains two cleavage sites, where cleavage is stimulated by SCC1 phosphorylation. Substrate recognition and cleavage are only partly understood. Here, we determined structures of human separase in apo- or substrate-bound forms that, together with biochemical analysis, provide critical insights into separase cleavage regulation. We verify the first SCC1 cleavage site and reassign the second. We show that substrates, including separase autocleavage sites and the two SCC1 cleavage sites, interact with docking sites in separase, including five phosphate-binding sites. We also describe the interaction between the cohesin subunit SA1/SA2 and separase, which promotes cleavage at the second SCC1 site. Using cross-linking mass spectrometry and cryo–electron microscopy, we propose how cohesin is targeted by human separase. Our work provides an extensive functional and structural framework that explains a key event in cell division.

## Teaser

Structural studies reveal how human separase binds the kleisin subunit SCC1 of cohesin to trigger sister chromatid separation.

## INTRODUCTION

**Fig. 1.**
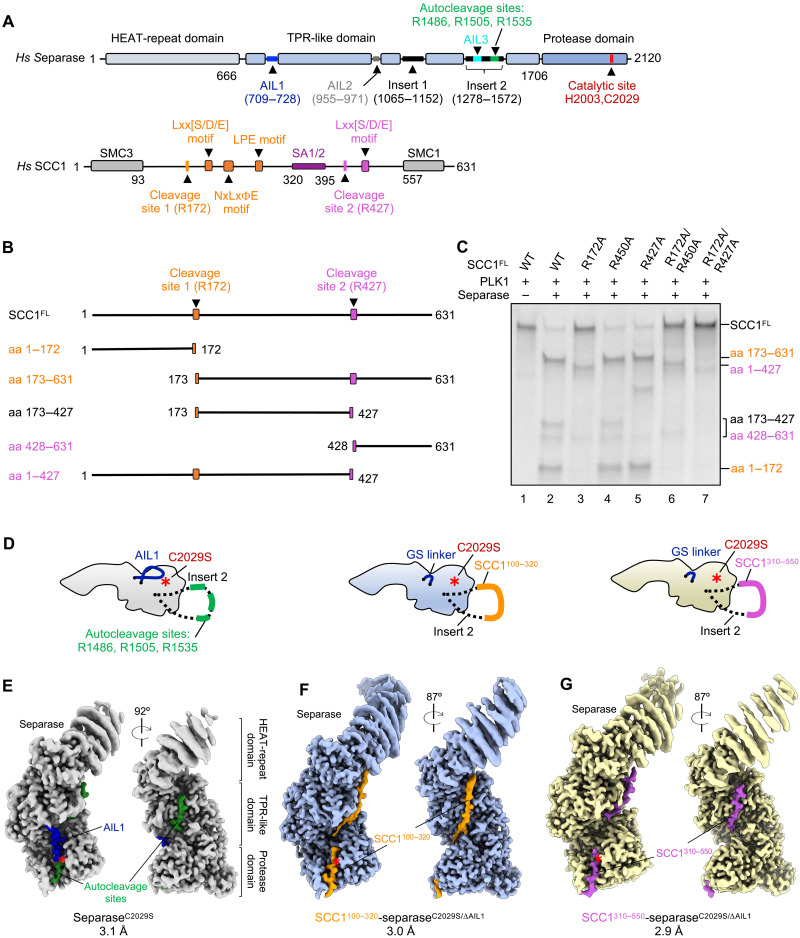
Cleavage of SCC1 at two sites and cryo-EM reconstructions of human separase in apo- or substrate-bound states. (**A**) Domain organization of human separase and SCC1. Hs, *Homo sapiens*. Separase consists of three domains shown as blocks: a HEAT-repeat domain (gray), a TPR-like domain (light blue), and a C-terminal protease domain (blue). The TPR-like domain contains autoinhibitory loops 1 to 3 (AIL1 to 3), shown with lines in dark blue, gray, and cyan, as well as two large flexible insertions, insert 1 (amino acids 1065 to 1152) and insert 2 (amino acids 1278 to 1572) illustrated in black. Three autocleavage sites (green line) are in the insert 2. The catalytic site consists of two conserved residues H2003 and C2029 (red). The N-terminal structural maintenance of chromosome 3 (SMC3)–binding domain and C-terminal SMC1-binding domain of SCC1 are indicated with gray blocks. SCC1 contains two cleavage sites: cleavage site 1 (R172) with nearby substrate motifs (orange) and cleavage site 2 (R427) with nearby substrate motifs (orchid). The SA1/2-binding region of SCC1 is shown in purple. (**B**) Schematic representation of SCC1 cleavage products resulting from two sites. aa, amino acids; FL, full-length. (**C**) Cleavage assay using ^35^S-labeled wild-type SCC1 and mutants as substrates. Results are representative of three independent experiments. WT, wild-type. (**D**) Fusion strategy for reconstitution of separase-substrate complexes. Left: Inactive apo-separase (separase^C2029S^; light gray) with insert 2 shown with a dashed line. AIL1 and autocleavage sites are color coded as in (A). Middle and right: Region containing three autocleavage sites in insert 2 is replaced by SCC1 fragments: SCC1 site 1 (amino acids 100 to 320: orange) or SCC1 site 2 (amino acids 310 to 550; orchid). AIL1 is replaced by a GS linker (ΔAIL1) (*8*). The C2029S mutation in separase indicates an inactive variant. Red star: the catalytic site. (**E** to **G**) Cryo-EM reconstructions of apo-separase [C2029S; (E)] and separase bound to SCC1 [amino acids 100 to 320; (F)] and SCC1 [amino acids 310 to 550; (G)]. The color codes are the same as in (D).

In early mitosis, the sister chromatids are linked by the protein complex cohesin (*1*–*3*). Chromosome segregation in anaphase is initiated by the proteolytic cleavage of the SCC1/RAD21 subunit of the cohesin ring by the cysteine protease separase (*4*–*7*). Human separase is a large protein (2120 amino acids) composed of three domains: an N-terminal HEAT-repeat domain, a central tetratricopeptide repeat (TPR)-like domain, and a C-terminal caspase-related protease domain (*8*, *9*). The central TPR-like domain contains two large unstructured inserts and several smaller loop regions, including three separase autocleavage sites (*10*, *11*) in insert 2 (Fig. 1A, top).

Because of its central function in cell cycle progression, separase activity is tightly controlled by multiple mechanisms. In vertebrates, activity is regulated through the concerted action of two mutually exclusive inhibitors: the universal inhibitor securin (*10*, *12*, *13*) and a heterotrimeric complex of cyclin-dependent kinase 1 (CDK1), its regulatory subunit CKS1 or CKS2, and the cyclin subunit B1 or B2 (the CCC complex) (*14*–*16*). Recent cryo–electron microscopy (cryo-EM) structures provided detailed insights into the mechanisms of human separase inhibition (*8*). Securin directly blocks substrate access to the active site and nearby docking sites by mimicking substrate binding, whereas the CCC complex rigidifies autoinhibitory loop (AIL) segments in separase that sterically preclude substrate binding. During metaphase, securin destruction leads to activation and autocleavage of separase. Autocleavage has been proposed to promote cyclin B–dependent regulation of separase (*17*), implying a handover mechanism for separase regulation (*9*, *17*).

Cleavage of separase substrates occurs at [D/E/S]ΦExxR motifs (P6 to P1 substrate positions, where cleavage occurs after the invariant R at the P1 position) (*4*, *7*, *18*). The structural basis of cleavage site binding at the active site was first revealed by the crystal structure of the separase protease domain from *Chaetomium thermophilum* fused to a short peptide mimicking the minimal SCC1 cleavage motif (*19*). Two cleavage sites have been described in human SCC1 (*7*, *20*). R172^SCC1^ is well established as cleavage site 1 (*7*, *8*, *21*). Site 2 is not as well understood, but it has been proposed (*20*) that cleavage occurs after R450^SCC1^.

Multiple ExxR sequences exist in SCC1 and across the proteome that are not cleaved, indicating the need for additional specificity determinants. Recently, docking sites outside the active site (exosites) were identified by biochemical and structural studies (*8*, *21*, *22*). SCC1 contains LPE and NHLEYE motifs that are required for efficient site 1 cleavage, and securin contains related motifs required for binding. The cryo-EM structure of the separase-CCC complex revealed that AILs dispersed throughout separase block substrate-binding sites. These lines of evidence suggest that substrates interact at a series of docking sites along the surface of separase, from the C-terminal active site to the N-terminal HEAT-repeat domain.

Cleavage of substrates by separase is often enhanced by substrate phosphorylation. Best understood is phosphorylation at the P6 position (*19*, *23*, *24*). The phosphoserine in the pSxExxR motif is ideally positioned to interact with a large basic patch next to the active site (*9*, *19*). However, there are other, more distant phosphorylation sites that stimulate cleavage. In SCC1, phosphorylation of multiple sites enhances cleavage (*20*). Cleavage of other substrates [Rec8, pericentrin (PCNT), and Slk19] is also stimulated by phosphorylation at multiple sites (*25*–*27*). However, we do not understand the structural basis for separase interaction with these phosphorylation sites.

We also do not know how separase interacts with the complete cohesin complex. Cohesin is a ring-shaped protein complex composed of four protein subunits (*28*): the structural maintenance of chromosome (SMC) proteins SMC1 and SMC3, the kleisin subunit SCC1, and the SCC3 subunit (SA1 or SA2 in mammals), which binds the disordered region of SCC1 between the two separase cleavage sites (*29*, *30*). It is conceivable that subunits other than SCC1 interact with separase to influence SCC1 cleavage.

We used a structural approach to deepen our understanding of substrate recognition and cleavage by human separase. Using cryo-EM structures of separase in apo- and substrate-bound forms, we define the human SCC1 cleavage sites and provide insights into the recognition of substrate motifs and phosphorylation sites distant from the cleavage site. We also describe an interaction between the cohesin subunit SA2 and separase, which promotes SCC1 cleavage at site 2. Last, we propose a model of separase binding to the cohesin ring.

## RESULTS

### Identification of separase cleavage sites in human SCC1

We first set out to corroborate the two described (*7*, *20*) cleavage sites in human SCC1 in vitro. We purified active separase and analyzed separase-mediated cleavage of radiolabeled wild-type and mutant forms of SCC1 in established cleavage assays (see Materials and Methods) (*8*, *21*). Because phosphorylation of SCC1 stimulates its cleavage by separase (*7*, *8*, *20*, *23*, *24*), the purified kinase domain of Polo-like kinase 1 (PLK1) (*31*) was routinely added to cleavage reactions.

We found that full-length phosphorylated SCC1 is efficiently cleaved by separase, resulting in a pattern that is consistent with cleavage at two sites (Fig. 1, B and C, lane 1 versus lane 2). Mutation of the previously established site 1, R172, to alanine resulted in a substantial decrease in SCC1 cleavage (Fig. 1C, lane 3). In contrast, mutation of R450, the second proposed cleavage site, did not affect cleavage (Fig. 1C, lane 2 versus lane 4) (*7*, *20*). To identify the second SCC1 cleavage site, we generated a series of ExxR mutants in SCC1 and found that mutation of R427 strongly impaired the generation of smaller cleavage products (amino acids 173 to 427 and 428 to 631) (Fig. 1, B and C, lane 5), although cleavage of full-length SCC1 appeared unaffected. A double mutant containing mutations of both R172 and R450 showed a phenotype identical to that of the R172-only mutant, whereas a R172 R427 double mutant was not cleaved by separase (Fig. 1C, lanes 6 and 7). These results suggest that the first cleavage site in human SCC1 is R172 as reported, but the second site is at R427 and not R450.

### Structure of inactive apo-separase reveals binding sites for autocleavage sites

Previous low-resolution studies suggested that the overall structure of separase is not affected by securin binding (*21*). However, a high-resolution structure of separase in the absence of bound inhibitor proteins has not yet been determined for any species (*8*, *19*, *32*, *33*). To gain further insights into autocleavage of human separase, we subjected purified wild-type and inactive mutant (catalytic cysteine replaced with serine: separase^C2029S^) apo-separase to cryo-EM analysis (Fig. 1, D and E, and figs. S1 and S2). Inactive separase is characterized by a single major band on SDS polyacrylamide gel electrophoresis (SDS-PAGE), whereas active separase autocleavage results in multiple distinct cleavage products (fig. S1, A and B). We determined the structures of active and inactive apo-separase at overall resolutions of 3.3 and 3.1 Å, respectively (figs. S1, I to L, and S2 and table S1). Separase consists of an N-terminal HEAT-repeat domain, a central TPR-like domain, and a C-terminal protease domain (Fig. 1, A and E). As observed in previous human separase structures (*8*), the N-terminal HEAT-repeat domain of separase moves as a flexible rigid body with respect to the rest of the protein (Fig. 1E and fig. S1, I and J). Overall, the structures of cleaved active and intact inactive apo-separase are indistinguishable, with a root mean square deviation of 0.524 Å over 1089 C_α_ atoms, confirming previous evidence that the autocleavage products of active separase remain tightly associated (*10*). The apo-separase structures are also almost identical to published human separase structures (*8*) bound to the inhibitor securin [Protein Data Bank (PDB): 7NJ1] or the CCC complex (PDB: 7NJ0) or when bound to the substrate SCC1 (this study; fig. S3 and table S2). Thus, the overall separase structure is not affected by interactions with inhibitors or substrates.

In the EM maps of inactive and active apo-separase, we observe clear densities for AIL1, which we identified in our previous studies of the separase-CCC complex (*8*). AIL1 is positioned in a substrate-binding cleft next to the catalytic site (Fig. 1, D and E, and fig. S4, A and B). Deletion of AIL1 increases separase activity, likely through increased substrate affinity (*8*). Furthermore, the high-affinity pseudosubstrate securin contains a motif that displaces AIL1. In the apo-separase structures, however, the densities for the AIL1 segments are weaker than those of the separase-CCC structure, probably due to a lower occupancy of AIL1 in this conformation in the apo-separase EM maps. It therefore seems likely that the AIL1 segment exhibits inherent flexibility and acts as a modulator of separase cleavage activity. When bound to native substrates or securin, the loop is displaced, which allows positioning of the binding partner in the active site pocket.

In addition to the density of AIL1, the EM map of inactive apo-separase (separase^C2029S^) contains other densities around the active site and at a conserved positively charged patch in the central TPR domain (Fig. 1E and fig. S4B). This density is absent in the EM maps of active apo-separase or inactive separase with the three autocleavage sites deleted (figs. S4A and S5), suggesting that these densities are related to autocleavage. The quality of the separase^C2029S^ map allows the unambiguous sequence assignment of these densities. The density around the active site represents the first autocleavage motif ^1481^GPEIMR^1486^, whereas the second additional density can be annotated to the third autocleavage motif ^1530^EWELLR^1535^ (P6 to P1 position with the ExxR cleavage motif in bold) (*8*). This second binding site on separase was previously predicted using AlphaFold2 (*34*) structure predictions of yeast separase in complex with SCC1 or Rec8 (*22*). It is likely that the first autocleavage site inserted into the catalytic pocket represents the primary cleavage site, but cleavage can also occur at the two other autocleavage sites (*10*, *11*). As we noted recently, the first autocleavage site of separase overlaps with a potential protein phosphatase 2A (PP2A) binding site, and autocleavage of separase abrogates PP2A binding (*9*, *35*). The separase-binding motifs are discussed in detail in a later section.

### Overall architecture of separase-SCC1 complexes

**Fig. 2.**
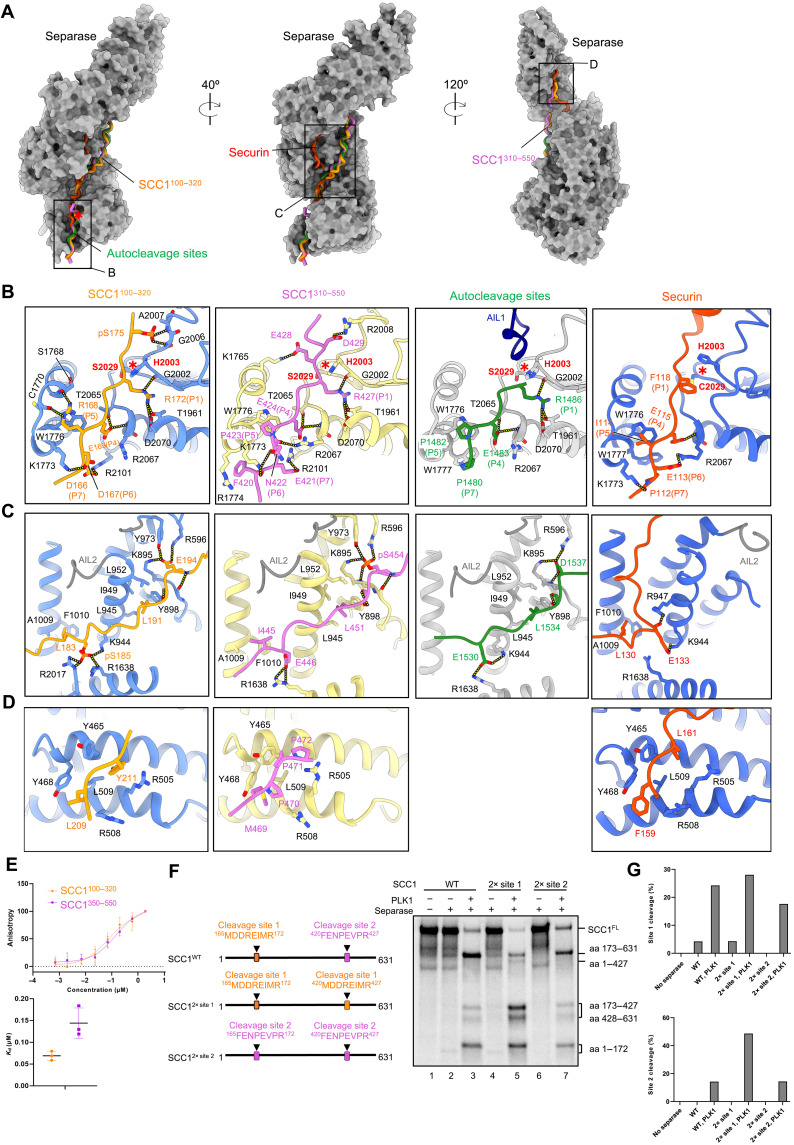
Multiple substrate motifs interact with separase. (**A**) Views of SCC1 site 1 (amino acids 100 to 320) and site 2 (amino acids 310 to 550), plus autocleavage sites and securin binding across the three domains of separase. Structures of separase^C2029S^, securin-separase complex (PDB: 7NJ1), and SCC1^310–550^-separase^C2029S/ΔAIL1^ are aligned to SCC1^100–320^-separase^C2029S/ΔAIL1^. Separase is depicted as surface representation in dark gray. SCC1 (orange or orchid), securin (orange red), and autocleavage sites (green) are shown as cartoon. (**B**) Close-up view of SCC1, securin, and autocleavage site binding near the catalytic site of separase. Hydrogen bonds are indicated with yellow dashed lines. (**C**) Close-up view of SCC1, securin, and autocleavage site binding to the TPR-like domain of separase. (**D**) Close-up view of SCC1 and securin binding to the HEAT-repeat domain of separase. (**E**) Affinity measurement of unphosphorylated SCC1^100–320^ and SCC1^350–550^ binding to separase^C2029S^ using fluorescence polarization. Each experiment was repeated three times; data points indicate mean ± SEM. (**F**) Cleavage assay of ^35^S-labeled wild-type SCC1 and mutants containing two copies of cleavage site 1 motif (SCC1^2× site 1^) or two copies of cleavage site 2 motif (SCC1^2× site 2^). Left: Schematic representation of wild-type SCC1 and mutants. Right: Autoradiograph of SCC1 cleavage. Results are representative of three independent experiments. (**G**) Top: Quantification of relative abundance of site 1 cleavage fragment (amino acids 1 to 172). Below: Quantification of relative abundance of site 2 cleavage fragments (amino acids 173 to 427 plus amino acids 428 to 631). For normalization, the intensities of the cleavage fragments were divided by the total intensities of all bands in the respective lanes.

We next sought to determine the structure of SCC1 bound to separase. Attempts to solve a separase-SCC1 structure using wild-type but inactive proteins were unsuccessful, even when full-length SCC1 was fused to the N terminus of separase and autocleavage sites in insert 2 were removed (fig. S5). We therefore designed new SCC1-separase^C2029S^ fusion constructs based on insights obtained from the structures of apo-separase, separase bound to the CCC complex, and the SCC1-separase fusion protein. To reduce the substrate-blocking effect of AIL1, we replaced this loop with a short flexible linker as previously described (*8*). We also replaced the autocleavage sites of insert 2, which otherwise compete with substrate binding, with defined SCC1 fragments containing either cleavage site 1 (SCC1^100–320^) or cleavage site 2 (SCC1^310–550^) (Fig. 1D, middle and right). We purified these SCC1-separase fusion constructs (fig. S6, A and B) and incubated them with PLK1 before cryo-EM analysis. We determined the structures of SCC1^100–320^ (site 1) or SCC1^310–550^ (site 2) bound to separase at overall resolutions of 3.0 and 2.9 Å, respectively (figs. S6, C to J, and S7 and table S1). The two structures show that each fragment binds similarly to separase using the same binding sites to zigzag across the surface of the enzyme in an antiparallel and extended form (Figs. 1, F and G, and 2A), reminiscent of the inhibitor securin (*8*, *32*, *33*).

While binding of the SCC1 fragments overlaps with securin around the catalytic site and the N-terminal HEAT-repeat domain of separase, the binding paths differ at the TPR-like domain (Fig. 2, A to D). Binding of securin to the TPR-like domain prompts a movement of AIL2 from its regular position (as observed in the apo-separase structure) to a binding site that is usually recognized by substrates. Securin therefore binds to different TPR-like repeats than substrates. Conversely, when in the apo- or substrate-bound state, AIL2 of separase adopts a conformation incompatible with securin binding. Therefore, AIL2 reinforces mutually exclusive binding of substrates or inhibitors where the binding paths do not overlap. The different binding modes likely result in different separase-binding affinities (fig. S3, B and C).

### Five positively charged docking sites anchor substrate binding to separase

The structure of cleavage site 1 bound to separase shows that R172 of SCC1 (P1 position) is deeply inserted into the catalytic pocket of separase, thereby orchestrating a configuration of the active site that allows substrate cleavage (Fig. 2B, SCC1^100–320^, and fig. S9C, bottom). A hydrogen-bonding network between R172^SCC1^ and residues lining the catalytic pocket, such as D2070, T1961, and G2002, facilitates a rotamer torsion of H2003^separase^ that, as a result, is optimally positioned for catalysis. This substrate-induced cleavage mechanism coordinated by an invariant arginine in substrates has been conserved in evolution (fig. S8A) (*19*, *32*, *36*). Substrate binding at the P1 to P6 positions near the active site is mediated by multiple interactions. R168^SCC1^ stacks onto W1776^separase^ at the P5 position and hydrogen bonds through its guanidinium group with the main chain oxygens of S1768^separase^ and C1770^separase^.

Critical electrostatic interactions form between D166^SCC1^ and E169^SCC1^ (P7 and P4 positions, respectively) and a positively charged patch immediately upstream of the separase active site. Key residues include R2067, R2071, and R2101 (Fig. 2B, SCC1^100–320^, and fig. S9C). This conserved positive patch on separase explains how negatively charged residues usually conserved at the P7 and the P6 substrate positions are recognized and how phosphorylation at the P6 position stimulates cleavage of SCC1 in budding yeast (*18*, *19*, *23*) and likely the human kinetochore protein Meikin (*37*). We refer to this patch as phosphate-binding site 1 (P-site 1).

Although human SCC1 is not phosphorylated at or near the P6 position (fig. S8A), phosphorylation elsewhere has been described to stimulate cleavage of site 1 (*20*). The structural basis of this effect is not known. In our structure of PLK1-phosphorylated SCC1^100–320^-separase^C2029S/ΔAIL1^, we found that serines 175, 185, and 189 of SCC1 are (partially) phosphorylated, according to EM densities and mass spectrometry (MS) (Fig. 2, B and C, and figs. S4, C, F, and G, and S9, A to D). Phosphorylated S175^SCC1^ (pS175^SCC1^) inserts into a pocket next to the active site, where it would sterically clash with the catalytic histidine H2003 in a securin-induced inactive rotamer conformation (fig. S10A). Thus, phosphorylation of S175^SCC1^ promotes the active rotamer conformation. The ideal positioning of H2003 is further supported by pS175^SCC1^ forming multiple hydrogen bonds with the side chain of R2008^separase^ and the main chain amino groups of G2004^separase^, A2005^separase^, and G2006^separase^, forming P-site 2 (Fig. 2B and fig. S9, B and C). The latter group of residues is in a loop segment adjacent to the catalytic histidine that is likely to be rigidified upon binding to pS175^SCC1^. As observed previously (*20*), S175A mildly reduces separase cleavage activity, and a S175E mutant shows slightly enhanced cleavage (fig. S10B).

The mild effects of S175 mutations suggested that additional SCC1 phosphosites stimulate separase cleavage activity. We observe a density for pS185 in our EM map (fig. S4, C and G). pS185^SCC1^ is recognized by a strongly positively charged P-site 3, created by K944, R1638, and R2017 at an interdomain cleft of separase (fig. S9D). L183^SCC1^ is inserted into an adjacent hydrophobic groove and stabilizes pS185 at the phosphate-binding site (Fig. 2C). This site is also bound by a previously described LPEE motif in securin (*21*), in which the leucine binds the hydrophobic groove, and the second glutamate is recognized by the positively charged site (Fig. 2C).

S189 phosphorylation was weakly detected in our MS data (fig. S9A) and is known from previous studies to be phosphorylated in vivo (*38*). However, phosphorylation at this site was not observed in our cryo-EM maps, but S189 is well positioned to interact with P-site 4 formed by lysine residues K944 and K1645 and presumably R901 (fig. S9D). As discussed below, P-site 4 is also positioned to interact with pS449 in the site 2 structure and E1532 in the autocleavage site structure (fig. S9D). We mutated the ^185^STTTS^189^ motif of SCC1 and studied its effects in combination with the S175A^SCC1^ mutation. The combination of multiple mutations noticeably reduced cleavage efficiency by separase, as shown previously (fig. S10C) (*39*).

The TPR-like domain contains P-site 5 that in the separase-securin complex structure is blocked by AIL2 (fig. S3, B and C). When SCC1 site 1 is bound, AIL2 is in an open conformation as in the apo-structure, enabling access to a positive patch recognized by E194^SCC1^ (Fig. 2C and fig. S9D). E194^SCC1^ hydrogen bonds with R596, K895, and Y973 of separase. A highly conserved leucine residue (L191^SCC1^), three amino acid N-terminal of E194^SCC1^, binds to a hydrophobic pocket and stacks onto Y898 of separase to serve as an additional anchor point. The resulting Lxx(S/D/E) motif is highly conserved in separase substrates (fig. S8B). It is also mimicked by AIL2 (L959 and D972) (fig. S3C). In our SCC1 site 2 structure, the acidic side chain is replaced by a phosphorylated serine as described below, and thus, P-site 5 represents a fifth phosphate-binding site.

Last, we also observe binding of an SCC1 fragment to the N-terminal HEAT-repeat domain of separase. This interaction is mediated by hydrophobic contacts: L209^SCC1^ and Y211^SCC1^ mediate stacking interactions with Y465^separase^ to tether SCC1 site 1 to the separase HEAT-repeat domain (Fig. 2D and fig. S4E). These residues are part of an NHLEYE motif (fig. S8C) that we described previously as an enhancer of SCC1 cleavage (*8*). Because of lower resolution in this map region, model building was aided by AlphaFold3 (AF3) predictions (*40*).

Our previous work showed that an LPE motif in SCC1 is required for site 1 cleavage in the absence of phosphorylation (*21*). As mentioned above, a similar LPEE motif in securin interacts with P-site 3 of separase (*21*). However, in the separase-SCC1 structure, we observe that the LPE motif is not involved in binding to P-site 3 when SCC1 is phosphorylated. We found that cleavage of phosphorylated site 1 in vitro does not require the LPE motif (fig. S9E).

Using the same fusion strategy, we determined the structure of separase bound to SCC1 cleavage site 2 (SCC1^310–550^-separase^C2029S/ΔAIL1^). Overall, the binding mode of the second cleavage site is analogous to that of site 1. R427 of SCC1 is inserted into the catalytic site pocket, consistent with our biochemical identification of R427^SCC1^ as the second cleavage site (Fig. 2B, SCC1^310–550^, and fig. S9C). E421^SCC1^ and E424^SCC1^ (P7 and P4 positions, respectively) are recognized by residues forming P-site 1 (R2067, R2071, and R2101), whereas W1776^separase^ forms stacking interactions with P423^SCC1^ at the P5 position. In addition, F420^SCC1^ stacks onto R1774^separase^. The pS175 described for site 1 is substituted with D429, which possibly hydrogen bonds with R2008^separase^, part of the loop that harbors the catalytic H2003^separase^ (Fig. 2B, SCC1^310–550^). Similarly, pS185 in site 1 is replaced with a glutamate, E446^SCC1^, which binds to the basic residues of P-site 3 (K944, R947, R1638, and K1645). Another similarity between site 1 and site 2 is that E446^SCC1^ binding to P-site 3 is supported by a hydrophobic N-terminal anchor: I445^SCC1^ binds to the adjacent hydrophobic groove, thereby stabilizing and precisely positioning E446^SCC1^.

P-site 4 binds to S449 (fig. S9D), a residue that is heavily phosphorylated in our MS data (fig. S9A) and in previous studies (*38*). Thus, as mentioned above, P-site 4 provides a likely binding site for phosphoserines in cleavage sites 1 and 2. Because phosphorylation of S189 or S449 was not seen in our cryo-EM density maps, we modeled the recognition of pS449 in fig. S9D.

While P-sites 2 and 3 recognize phosphoserines in cleavage site 1 but not in site 2, the situation is reversed for P-site 5. Here, as described above, site 1 binds to P-site 5 through E194, whereas at site 2, we observe a phosphoserine bound to P-site 5 (Fig. 2C, SCC1^310–550^, and fig. S9, B and D). The phosphate group of pS454^SCC1^ forms multiple hydrogen bonds with R596, K895, and Y973 of separase. Individual or combined mutations of I444^SCC1^, E446^SCC1^, L451^SCC1^, or S454^SCC1^ to alanines impair the generation of site 2 cleavage products in vitro, indicative of reduced binding of SCC1 to separase (fig. S10C). These results are consistent with previous evidence that a S454A mutation inhibits cleavage at the second site (*20*).

Comparable to site 1, SCC1 also binds via site 2 to the N-terminal HEAT-repeat domain. M468 and P470 of a conserved MPPP motif (amino acids 468 to 471) tether SCC1 to the HEAT-repeat domain (fig. S8C). Here, M468^SCC1^ binds separase as a hydrophobic anchor like L209^SCC1^ of site 1. P470^SCC1^ of site 2 forms stacking interaction with Y468^separase^ analogous to Y211^SCC1^ of site 1 (Fig. 2D, SCC1^100–320^ and SCC1^310–550^, and fig. S4E). This binding mode is mimicked by securin. F159^securin^ and L161^securin^ bind to the same HEAT repeat by hydrophobic and stacking interactions, respectively (Fig. 2D, securin, and fig. S8D) (*21*).

With SCC1 structures in mind, we can now understand in more detail the binding of the first autocleavage site in our structure of inactive apo-separase. In this structure, R1486^separase^ is inserted into the active site, priming the catalytic dyad in a conformation suitable for substrate cleavage. For the first autocleavage site, only E1483^separase^ at the P4 position is recognized by P-site 1. At the P5 and P7 positions, two proline residues, P1482^separase^ and P1480^separase^, form stacking interactions with the highly conserved W1776^separase^ and W1777^separase^, respectively (Fig. 2B, autocleavage sites). The prolines are interspersed by G1481^separase^. Therefore, in this binding mode, the usually negatively charged P6 substrate position that is recognized by P-site 1 is replaced by G1481^separase^, which provides a flexible hinge to enable stacking interactions of P1482^separase^ with W1777^separase^. This different binding mode might explain the sequence variability of the substrate binding motif at the P6 position (fig. S8A). Another feature that discriminates binding of the autocleavage site from substrates or securin binding is that AIL1 is not displaced by the autocleavage motif. As a result, AIL1 is present in our EM map and the derived model (Figs. 1E and 2B, autocleavage sites). The molecular interactions between E1530^separase^ and P-site 3 are reminiscent of E446^SCC1^ of SCC1 site 2. Binding to P-site 4 differs between the autocleavage site motif and the SCC1 cleavage sites. In SCC1, phosphoserine residues are likely to be recognized by P-site 4, whereas in the autocleavage motif, a glutamate (E1532) mimics substrate phosphorylation (fig. S9D). Further downstream, an LxxD motif binds to P-site 5 as observed for the LxxE motif of site 1 or the LxxpS motif of site 2 (Fig. 2C).

Comparing the binding modes of SCC1 cleavage sites 1 and 2 reveals not only commonalities but also differences in their recognition by separase (movie S1), which might result in different binding affinities. In addition, we observe a strong preferential cleavage of site 1 in our cleavage assays (fig. S10D). To estimate binding affinities for the two sites separately, we purified two SCC1 fragments containing either cleavage site 1 or 2 (SCC1^100–320^ and SCC1^350–550^). Both fragments were N-terminally linked to the maltose-binding protein (MBP), and a tetracysteine peptide was fused to the C terminus for binding by a specific fluorophore [fluorescein arsenical hairpin (FlAsH)–1,2-ethanedithiol (EDT_2_)]. This setup allowed us to perform fluorescence polarization experiments. Binding of inactive separase to SCC1 causes a slowed rotation (higher anisotropy) of the fluorescently labeled SCC1 due to a substantial mass increase. Our assay shows that the SCC1^100–320^ construct exhibits a roughly threefold lower dissociation constant (*K*_d_) than the SCC1^350–550^ construct (Fig. 2E), when both proteins are unphosphorylated. Phosphorylation of SCC1^100–320^ leads to an increase in binding affinity, indicated by a roughly twofold lower *K*_d_ compared to unphosphorylated SCC1^100–320^ (fig. S9G).

To reveal the effect of the cleavage motif alone (P8 to P1 positions) on cleavage activity, independent of other binding sites, we designed two artificial SCC1 constructs. In an SCC1^2×site1^ construct, cleavage site 2 was substituted by site 1 to generate a construct containing two copies of site 1. Conversely, we replaced cleavage site 1 with site 2 to generate a construct containing two copies of site 2 (SCC1^2×site2^) (Fig. 2F). As we observed previously, phosphorylation of wild-type SCC1 strongly stimulates cleavage (Fig. 2, F and G). SCC1^2×site1^ is more efficiently cleaved than wild-type SCC1, resulting in enhanced generation of smaller cleavage products (Fig. 2F; compare lane 3 versus lane 5). In contrast, SCC1^2×site2^ is less efficiently cleaved than wild-type SCC1 or SCC1^2×site1^ (Fig. 2, F and G). As expected, if enhanced binding affinity is caused by increased electrostatic interactions upon substrate phosphorylation, then cleavage efficiency declines at increasing salt concentrations (fig. S9F). These experiments with swapped cleavage motifs indicate that although multiple docking motifs are recognized by separase, specific cleavage site motifs are favored.

### The cohesin subunit SA1 or SA2 stimulates SCC1 cleavage at the second cleavage site

**Fig. 3.**
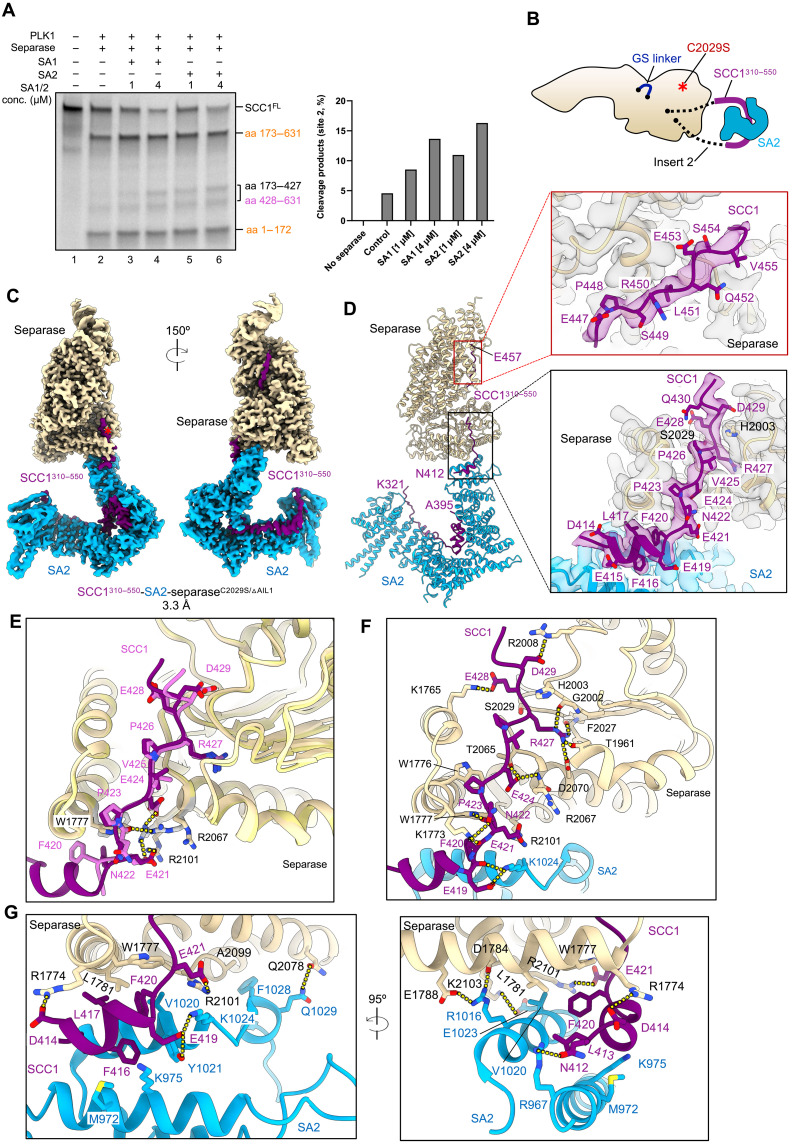
SA1/2 stimulates SCC1 cleavage at site 2 and cryo-EM structure of separase bound to SCC1-SA2 complex. (**A**) Cleavage assay showing stimulation of SCC1 cleavage at site 2 by SA1/2 proteins, tested at two different concentrations (conc.,1 and 4 μM). conc., concentration. Left: Autoradiograph showing cleavage products of ^35^S-labeled SCC1. Right: Quantification of relative abundance of site 2 cleavage fragments (amino acids 173 to 427 and 428 to 631). For normalization, the intensities of the site 2 cleavage fragments were divided by the total intensities of all bands in the respective lanes. Results are representative of three independent experiments. (**B**) Schematic representation of the SCC1-SA2-separase complex reconstitution. SCC1 (amino acids 310 to 550) fragment (purple) includes the SA2 binding region and cleavage site 2. SA2 is shown in deep sky blue, and separase is depicted in wheat. (**C**) Views of the cryo-EM map of the SCC1^310–550^-SA2-separase^C2029S/ΔAIL1^ complex. (**D**) Ribbon representation of the SCC1^310–550^-SA2-separase^C2029S/ΔAIL1^ complex highlighting the interaction interfaces between the three proteins. Red box: cryo-EM density showing the interaction interface between SCC1 and the TPR-like domain of separase. Black box: cryo-EM density showing the interaction interface between SCC1, SA2, and the protease domain of separase. (**E**) Close-up view showing SCC1 binding to the protease domain of separase in the complexes of SCC1^310–550^-SA2-separase^C2029S/ΔAIL1^ and SCC1^310–550^-separase^C2029S/ΔAIL1^. The two structures were aligned using separase as a reference. SCC1 in the two complexes, depicted as sticks, is shown in purple and orchid. Side chains of W1777, R2067, and R2101 in the two complexes are shown in wheat and gray, respectively. Hydrogen bonds are indicated with yellow dashed lines. (**F**) Close-up view of the cleavage site 2 motif of SCC1 binding near the catalytic site of separase. (**G**) Two views of the interaction interface formed by a short helix of SCC1 (amino acids 413 to 419), C-terminal helices of SA2, and the protease domain of separase.

The long disordered central region of SCC1 includes a binding site for the cohesin SA1/2 subunit (Fig. 1A, bottom) (*41*). Given the proximity of the SA1/2 binding site to site 2, we speculated that SA1/2 might affect separase cleavage activity. Consistent with this possibility, we found that preincubation of SCC1 with SA1 or SA2 stimulates the generation of smaller cleavage products while not affecting larger cleavage products (Fig. 3A and fig. S11A). SA2 stimulates cleavage of SCC1 site 2, but not site 1, even in the absence of PLK1 (fig. S11A).

To explore these effects in detail, we prepared a complex composed of SCC1^310–550^-separase^C2029S/ΔAIL1^ and full-length SA2 (Fig. 3B). The proteins formed a stable complex in vitro that was subjected to structural studies without prior phosphorylation (fig. S12A). In our cryo-EM analysis, we observed multiple classes—a ternary complex consisting of SCC1^310–550^-separase^C2029S/ΔAIL1^ and full-length SA2, SCC1^310–550^-separase^C2029S/ΔAIL1^, and SCC1^310–550^ bound to SA2—and determined their structures to resolutions of 3.4, 2.8, and 2.9 Å, respectively (fig. S12, B to J, and table S1). Because of the heterogeneity of the sample, likely caused by low-affinity binding between SA2 and separase, and due to preferential orientation of particles, we collected more than 50,000 micrographs to obtain high-resolution structures. The complex processing scheme is depicted in fig. S13.

The SCC1^310–550^-SA2-separase^C2029S/ΔAIL1^ complex is highly elongated and measures more than 250 Å in its longest and less than 50 Å in its shortest dimension [the flexible N-terminal HEAT-repeat domain of separase is not shown in Fig. 3 (C and D)]. The C-terminal HEAT repeat of the hook-shaped SA2 protein packs onto helices that surround the active site of separase to form a minimal protein-protein interface that is driven mainly by electrostatic interactions. For example, R1016^SA2^ forms hydrogen bonds with E1788^separase^ and D1784^separase^, and E1023^SA2^ interacts with K2103^separase^ (Fig. 3, C and G). This interface provides additional anchor points for SCC1 to be ideally positioned in and around the catalytic site (Fig. 3D and movie S2).

SCC1 site 2 binding to the TPR-like domain is virtually identical in the absence or presence of SA2, although we did not phosphorylate SCC1 with PLK1 in this sample (Fig. 3D and fig. S11B). However, simultaneous binding of SCC1 and SA2 to the protease domain of separase causes a reorganization of several side chains involved in the interaction of P-site 1 with the P6 substrate position. This interaction includes R2067^separase^, R2071^separase^, and R2101^separase^ and the highly conserved W1777^separase^ (Fig. 3E and fig. S11C). W1777^separase^ flips to a rotamer configuration that allows stacking interactions with F420^SCC1^. In the absence of SA2 binding, F420^SCC1^ is likely solvent exposed and flexible. Similarly, R1774^separase^ shifts by about 3 Å compared to the SCC1^310–550^-separase^C2029S/ΔAIL1^ structure. This side chain position facilitates hydrogen bonding between R1774^separase^ and D414^SCC1^, as well as the main chain oxygen of E415^SCC1^. The extensive hydrogen-bonding network that ensures correct positioning of the substrate arginine in the catalytic site is illustrated in Fig. 3F, which includes a short amphipathic helix of SCC1^413–419^ (fig. S11D). Additional interactions that stabilize a short SCC1 α helix at this interface of the separase protease domain and SA2 are between F416^SCC1^, M972^SA2^, and K975^SA2^, as well as hydrogen bonding between E419^SCC1^ and K1024^SA2^ (Fig. 3G). The overall structure of SA2 bound to separase and SCC1 is highly similar to the binary complex composed of SCC1^310–550^ and SA2 (fig. S11E). In line with our biochemical studies that demonstrate that both SA proteins can stimulate SCC1 site 2 cleavage by separase, the residues forming the interface between SA2 and separase are largely conserved in SA1 (fig. S11, F and G).

### The separase-cohesin complex structure

**Fig. 4.**
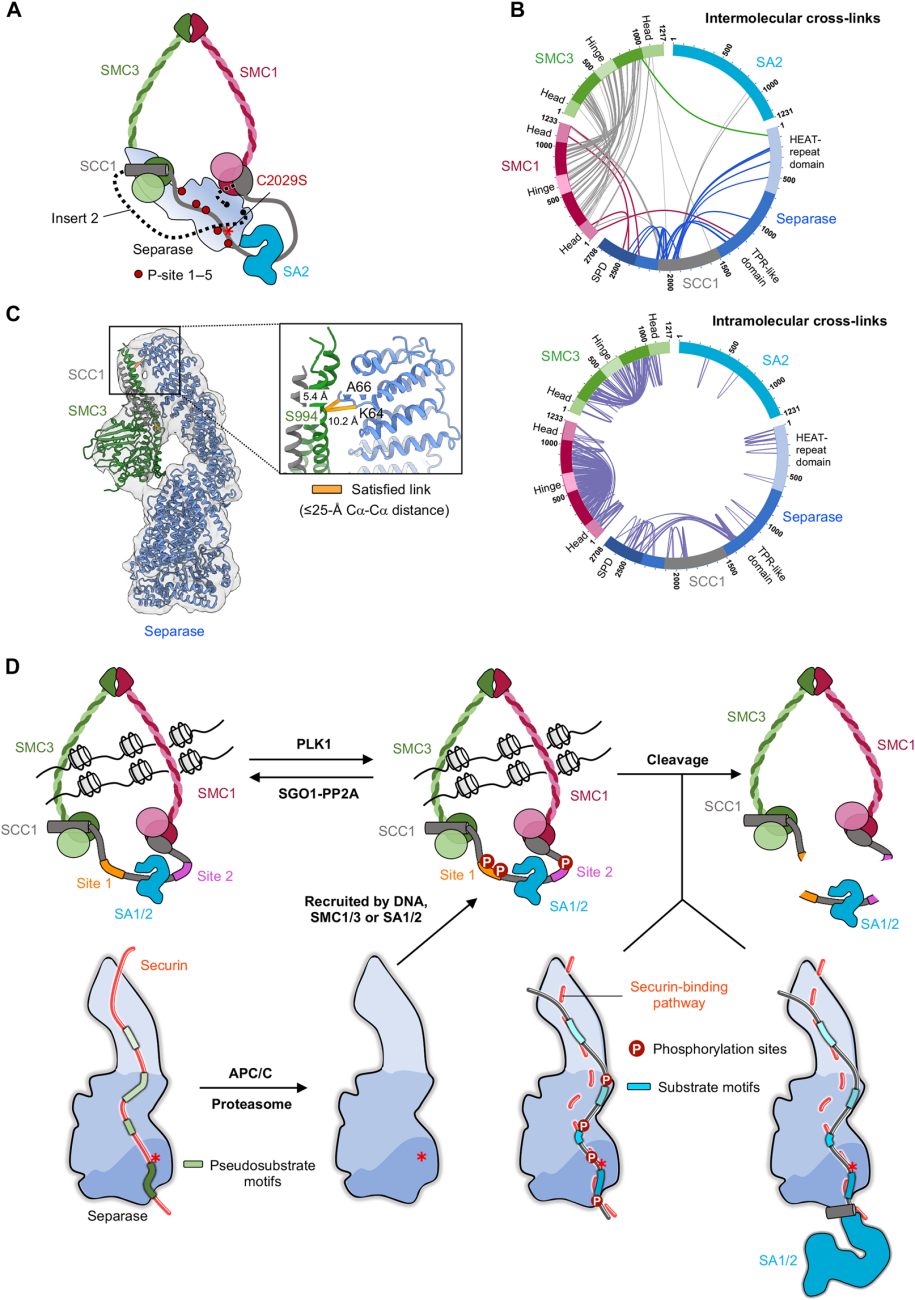
Cohesin targeting by separase is mediated by SMC subunits. (**A**) Schematic representation of the strategy for reconstituting the cohesin-separase complex. Full-length SCC1 replaces the autocleavage sites in insert 2 (dashed line) of separase. SMC3 is colored in light and forest green, SMC1 in light and dark red, SCC1 in gray, SA2 in deep sky blue, and separase in a blue gradient. P-sites 1 to 5 are indicated as red dots. (**B**) Cross-linking MS (XL-MS) analysis of cohesin-separase complex cross-linked using sulfosuccinimidyl 4,4′-azipentanoate (Sulfo-SDA). Circular view showing intermolecular cross-links (top) and intramolecular cross-links (bottom) in the cohesin-separase complex. Each protein is color coded as in (A). Cross-links between the cohesin subunits (SMC1, SMC3, and SCC1) and separase are shown with dark red, green, and blue lines, respectively. Intramolecular cross-links are shown in purple. (**C**) Cryo-EM map and model of separase bound to SMC3-SCC1. Satisfied Sulfo-SDA cross-links with Cα-Cα distances ≤ 25 Å are shown as orange solid lines. (**D**) A model illustrating phosphorylation- or SA1/2-dependent regulation and the specificity determinants of cohesin cleavage by separase. APC/C, anaphase-promoting complex/cyclosome.

To gain further insights into separase substrate recognition, we reconstituted a separase-cohesin complex using a protein engineering strategy like the one described above. Full-length SCC1 was inserted in place of the autocleavage sites of inactive separase, and AIL1 was deleted to enhance substrate binding affinity. We coexpressed the cohesin subunits SA2 and the SMC proteins SMC1 and SMC3 together with the SCC1-separase fusion construct and reconstituted the separase-cohesin complex in vitro (Fig. 4A and fig. S14A). A DNA fragment was added to the preparation to enhance complex formation. However, initial EM analysis did not yield promising separase-cohesin two-dimensional (2D) classes, and therefore, we stabilized the complex using cross-linking reagents (fig. S14B).

We used the highly specific photoreactive cross-linker sulfosuccinimidyl 4,4′-azipentanoate (Sulfo-SDA) to perform cross-linking MS (XL-MS) analysis. As expected, we observed numerous intra- and intermolecular cross-links between the adenosine triphosphatase head domains and the long coiled-coil regions of SMC1 and SMC3 proteins (Fig. 4B and fig. S14E). We also detected several reproducible cross-links between the SMC1 head domains and the C-terminal half of separase, as well as cross-links between the SMC3 head domains and the N terminus of separase (Fig. 4, B and C). While most of the observed cross-links are consistent with a Cα-Cα distance of 25 Å or less (satisfied links) and agree with our model, there are clusters of cross-links that exceeded 25 Å (long-distance links), indicating structural heterogeneity and/or flexibility of the complex.

We next used negative-staining EM to analyze a bis(sulfosuccinimidyl)suberate (BS3)–cross-linked sample. We detected three distinct complexes: the cohesin complex, separase bound to SA2, and separase bound to the head domains of SMC1 and/or SMC3 (fig. S14, C and D). All separase-SMC1-SMC3 complex particles were then subjected to further 3D classification, which resulted in three different classes that showed additional densities besides the unambiguous separase density (fig. S14, F to H). Class 1 and class 2 showed additional density near the C-terminal half of separase, and the size of this extra density corresponded well to the SMC1 head domains. Combined with the evidence from XL-MS for cross-links between SMC1 head domains and separase, we placed the SMC1 head domains in the C-terminal extra density (fig. S14, F and G). The third class showed two extra densities: the C-terminal extra density plus an additional density of similar size near the separase N terminus. Because we detected cross-link pairs between a helix protruding from the C-terminal head domain of SMC3 and the very N terminus of separase, we placed the head domains of SMC3 in this second EM density (fig. S14H).

We then subjected the cross-linked sample to cryo-EM studies and resolved the complex structure at an overall resolution of 3.5 Å (fig. S15). Again, we observed an extra density bound to the N-terminal HEAT-repeat domain (figs. S14H and S15D). Using a mask on the N-terminal HEAT-repeat domain to select for particles with the SMC3 protein bound resulted in 26,747 particles that refined to an overall resolution of about 4.3 Å. However, the local resolution of the extra density next to the N-terminal domain is lower, indicating a high degree of flexibility of the complex (fig. S15D). We fit our AF3-predicted model of the C-terminal SMC3 head domain in complex with SCC1 into this density, and this fit is mostly in agreement with our detected cross-links (Fig. 4C).

Integrating all available data allows us to propose a model in which separase not only binds to the substrate SCC1 but also recognizes the head domains of SMC1 and SMC3. In the case of SCC1 site 2 binding to separase, cleavage is further stimulated by an interaction with the SA1/2 subunit of cohesin, thereby providing additional SCC1 binding sites near the active site of separase (Fig. 4A).

## DISCUSSION

Our studies reveal that separase substrates bind in an extended antiparallel orientation to a series of docking sites along the surface of separase, from its C-terminal active site to its N-terminal HEAT-repeat domain (Fig. 4D). These docking interactions enhance substrate affinity and specificity, focusing the enzyme on a very small number of specific cleavage sites among the many ExxR motifs that exist in the proteome. About 20% of proteins in the budding yeast proteome carry an ExxR motif, but so far, only the cohesin subunits SCC1 and Rec8, and the kinetochore-associated protein Slk19, have been confirmed as separase substrates (*18*). Slk19 is cleaved after a DxxR motif, highlighting the sequence variability of the cleavage site motif.

The specificity of substrate binding is likely explained by the presence of multiple positively charged substrate-docking sites on separase that can interact either with acidic side chains or with phosphorylated substrate residues (table S3). Interactions between phosphorylated side chains and these sites explain previous evidence that phosphorylation promotes cleavage of sites 1 and 2 in SCC1. We suspect that these phosphate-binding sites are also involved in the recognition of other substrates, such as Rec8, PCNT, or Slk19, whose cleavage is promoted by multisite phosphorylation (*25*–*27*).

Our studies of substrate binding, together with our previous analysis of the separase-securin complex, demonstrate that substrates and inhibitors use a diverse array of modular sequence motifs to bind separase with high affinity and specificity. The ability of separase to interact with many different motifs allows interesting variations in the motifs used by different binding partners and even by the same partner. For example, as mentioned above, the five positively charged docking sites interact with phosphate in some cases and acidic residues in others, and P-site 1 can also accommodate other amino acids through a distinct binding mode. SCC1 site 1 can bind through two distinct mechanisms: Unphosphorylated SCC1 depends on LPE and NHLEYE motifs for efficient cleavage of site 1 in vitro and in vivo (*21*), but cleavage of phosphorylated SCC1 at site 1 does not require these motifs but instead depends on phosphorylated residues in SCC1. We further identify an important second motif, LxxpS/D/E, that is crucial for the binding of both SCC1 cleavage sites and the separase autocleavage site. We conclude that the high affinity of substrate and securin binding depends on a diverse selection of multiple low-affinity sites, each of which can be modified slightly without greatly affecting overall affinity. Searches for combinations of these motifs across the proteome might lead to the identification of previously unknown substrates.

It is critical for the success of mitosis that separase exhibits extreme specificity for a small group of substrates and avoids off-target cleavage of other proteins. One solution to this problem is that separase exhibits extremely low catalytic activity toward a short peptide substrate containing only the site 1 cleavage sequence (*21*). Cleavage at a substantial rate requires the boost in affinity and activity that results from highly specific interactions between separase and its targets. These interactions depend on the binding of substrate sequence motifs, but it seems likely that additional mechanisms focus the enzyme on the correct sites. We show, for example, that specificity for site 2 is enhanced by an interaction between separase and the cohesin subunit SA1/2. Additional specificity for cohesin might arise from the ability of separase to interact with DNA (*42*). Last, our studies of a separase complex with complete cohesin complex suggest that additional interactions between separase and the SMC subunits are also involved. The binding of the SMC head domains (from SMC1 and SMC3) might play an important role in cohesin recognition by interacting with separase on the opposite side from SCC1. These and other mechanisms ensure that the potentially dangerous protease activity of separase is focused precisely on the correct targets, resulting in a successful completion of cell division. These mechanisms might also be exploited by future targeted drug design studies. It is, for example, conceivable that down-regulating separase activity in specific cancer types could be facilitated by a specific inhibitor cocktail that targets multiple substrate-docking sites on separase (*43*), such as the P-sites 1 to 5.

## MATERIALS AND METHODS

### Expression vector construction

The cDNAs encoding human separase, SMC1, SMC3, and STAG1/2 (SA1/SA2) were synthesized as gene-optimized versions for expression in *Spodoptera frugiperda* (Sf9) cells. The cDNA for SCC1 was polymerase chain reaction (PCR) amplified from a human cDNA library. The apo-separase plasmid was generated by subcloning synthesized cDNA (Thermo Fisher Scientific) of separase (amino acids 1 to 2120) into a pF1 vector with a Twin-StrepII tag at the C terminus and a 3xFLAG tag at the N terminus. The securin^160–202^-separase fusion construct was described previously (*8*, *21*). SCC1-separase fusion constructs were generated by replacing the autocleavage fragment (amino acids 1482 to 1536) in insert 2 of separase with SCC1 fragments: amino acids 100 to 320 (site 1), amino acids 310 to 550 (site 2), or amino acids 1 to 631 (full length). In early studies, the C terminus of SCC1 (amino acids 1 to 631) was fused to the N terminus of separase through a long unstructured linker. SA1/2 subunits were cloned into the pF1 vector with a Twin-StrepII tag at the C terminus. SMC1 and SMC3 subunits were cloned into the pF1 vector harboring a dual promoter, with a Twin-StrepII tag at the C terminus of SMC1 and an 8xHis tag at the C terminus of SMC3. For fluorescence polarization experiments, SCC1 (amino acids 100 to 320) and SCC1 (amino acids 350 to 550) were cloned into a pETM41 vector containing an N-terminal 6xHis-MBP tag and a C-terminal tetracysteine FCM motif (FLNCCPGCCMEP) for FlAsH labeling (*44*). The longer SCC1 fragment (amino acids 100 to 550) was cloned into the pETM41 vector with a His-MBP tag at the N terminus. The kinase domain of the human PLK1 (amino acids 37 to 338) in the pETM41 vector was provided by C. Alfieri (ICR, London). All deletions and mutations were generated by mutagenesis PCR.

### Expression and protein purification

Human separase constructs and cohesin subunits were expressed with a baculovirus expression system. Typically, 25 ml of recombinant P3 baculoviruses were used to infect 500 ml of Sf9 insect cells (Invitrogen) at a cell density of 2.0 × 10^6^ to 3.0 × 10^6^ cells/ml. Infected cells were incubated at 27°C with shaking at 100 rpm for ~48 hours. Cells were harvested at a cell viability rate of 80 to 90%, flash frozen in liquid nitrogen, and stored at −80°C until further use. SCC1 constructs (amino acids 100 to 320, 350 to 550, and 100 to 550) and PLK1 (amino acids 37 to 338) were expressed in *Escherichia coli* BL21 cells at 18°C for 16 hours after induction with 0.5 mM isopropyl-β-d-thiogalactopyranoside.

Purification of all proteins and protein complexes was performed at 4°C. Cell pellets of 4-liter Sf9 cultures expressing apo-separase or SCC1-separase fusion complexes were resuspended in lysis buffer [50 mM Hepes-KOH (pH 8.0), 500 mM KCl, 5% glycerol, 0.5 mM EDTA, and 0.5 mM tris(2-carboxyethyl)phosphine (TCEP)] supplemented with protease inhibitor cocktail tablets (cOmplete EDTA-free, Roche Diagnostics GmbH) and SuperNuclease (5 units/ml; Sino Biological). The resuspended cells were lysed by sonication and centrifuged at 18,000 rpm for 1 hour. The clarified lysate was slowly (flow rate: 0.8 ml/min) loaded onto two tandem 5-ml Strep-Tactin Superflow Cartridge (QIAGEN), and the columns were washed with the lysis buffer until stable ultraviolet (UV) absorption was observed. Proteins were eluted in lysis buffer containing 2.5 mM d-desthiobiotin. The eluate was diluted with salt-free buffer to a final KCl concentration of 150 mM and loaded onto a 5-ml HiTrap Heparin HP column (Cytiva) equilibrated in a buffer of 50 mM Hepes-KOH (pH 8.0), 150 mM KCl, 5% glycerol, 0.5 mM EDTA, and 0.5 mM TCEP. Separase proteins were eluted using a linear gradient of KCl from 150 mM to 1 M. Peak fractions from the Heparin column were pooled, concentrated, and passed through a Superose 6 Increase 10/300 GL column (GE HealthCare Life Sciences), preequilibrated in gel filtration buffer [20 mM Hepes-KOH (pH 8.0), 300 mM KCl, and 0.5 mM TCEP]. The purified proteins were pooled, concentrated, flash frozen, and stored at −80°C for further use. Purification of securin^160–202^-separase fusion protein was performed as described previously (*8*, *21*), and the protein was stored in a buffer containing 20 mM Hepes-KOH (pH 8.0), 100 mM KCl, 10 mM MgCl_2_, 0.5 mM TCEP, and 5% glycerol.

SA1 and SA2 were purified using Strep-Tactin Superflow Cartridge (QIAGEN) columns. The lysis and washing buffer consisted of 50 mM Hepes-KOH (pH 8.0), 500 mM KCl, 5% glycerol, and 0.5 mM TCEP. The columns were extensively washed until a stable UV absorption was reached. SA1/2 proteins were eluted using wash buffer supplemented with 2.5 mM d-desthiobiotin. Fractions containing SA1/2 were concentrated and loaded on a Superose 6 Increase 10/300 GL column (GE HealthCare Life Sciences). The purified SA1/2 proteins were stored in gel filtration buffer [20 mM Hepes-KOH (pH 8.0), 300 mM KCl, and 0.5 mM TCEP] at −80°C. For expression of the SCC1^310–550^-SA2-separase^C2029S/ΔAIL1^ complex, recombinant P3 baculoviruses of SCC1^310–550^-separase^C2029S/ΔAIL1^ fusion construct and SA2 were used to coinfect 3 liters of Sf9 cells. The SCC1^310–550^-SA2-separase^C2029S/ΔAIL1^ complex was purified as described for SA1/2 proteins.

The cohesin-separase fusion complex was expressed in Sf9 cells by coinfection with recombinant P3 baculoviruses of SMC1-SMC3 subunits, the SCC1^1–631^-separase^C2029S/ΔAIL1^ fusion construct, and the SA2 subunit. Pellets of 5-liter Sf9 cells were lysed by sonication in lysis buffer [50 mM Hepes-KOH (pH 8.0), 300 mM KCl, 5% glycerol, 0.5 mM EDTA, and 0.5 mM TCEP] supplemented with protease inhibitors and SuperNuclease. The lysate was centrifuged at 18,000 rpm for 1 hour. The supernatant was loaded onto Strep-Tactin Superflow Cartridge (QIAGEN) columns at a flow rate of 0.8 ml/min, and proteins were eluted in lysis buffer containing 2.5 mM d-desthiobiotin. KCl concentration of the eluate was diluted to 150 mM, and the complex was further purified using a 5-ml HiTrap Heparin HP column (Cytiva). Peak fractions containing cohesin-separase fusion complexes were pooled, concentrated, and loaded on a Superose 6 Increase 10/300 GL column (GE HealthCare Life Sciences), preequilibrated in gel filtration buffer [20 mM Hepes-KOH (pH 8.0), 300 mM KCl, and 0.5 mM TCEP]. The purified proteins were concentrated to ~3.2 mg/ml, flash frozen, and stored at −80°C.

MBP-tagged SCC1 fragments (amino acids 100 to 320, 350 to 550, and 100 to 550) were purified using a HiTrap MBP column. Briefly, *E. coli* cells expressing SCC1 fragments were resuspended in lysis buffer [25 mM Hepes (pH 7.7), 500 mM NaCl, 1 mM EDTA, and 2 mM dithiothreitol (DTT)]. After centrifugation, the supernatant was filtered and applied to a 5-ml HiTrap MBP column equilibrated in lysis buffer. Proteins were eluted using a buffer containing 25 mM Hepes (pH 7.7), 150 mM NaCl, 10 mM maltose, and 2 mM DTT. Fractions containing SCC1 were pooled, concentrated to 1 ml, and loaded on a Superdex 200 Increase 10/300 GL gel filtration column (GE Healthcare Life Sciences) preequilibrated in 25 mM Hepes (pH 7.7), 150 mM NaCl, and 2 mM DTT. Purification of PLK1 was performed as previously described (*31*).

### Fluorescence anisotropy

SCC1 site 1 (amino acids 100 to 320) and SCC1 site 2 (amino acids 350 to 550) were labeled with the FlAsH-EDT2 fluorescent dye using a buffer containing 25 mM Hepes (pH 8.0), 150 mM NaCl, and 1 mM β-mercaptoethanol. The labeling reaction was initiated by mixing 5 μM protein with 25 μM dye, followed by an overnight incubation at 4°C. The excess dye in the samples was removed by gel filtration. Phosphorylated SCC1 site 1 (100 to 320) was generated by cleaving the MBP tag with TEV protease, followed by size exclusion chromatography to remove the free MBP protein, followed by 2 hours of incubation with PLK1 at 37°C at a molar ratio of roughly 8:1 (100 μg of SCC1 mixed with 9 μg of PLK1). A total of 5 mM adenosine 5′-triphosphate (ATP) and 10 mM Mg^2+^ was added to the reaction buffer.

Fluorescence anisotropy assays were performed to investigate the molecular interactions between separase and SCC1 site 1 (phosphorylated and unphosphorylated) or SCC1 site 2 (unphosphorylated). All measurements were conducted using a fluorescence spectrometer equipped with polarizers in both the excitation and emission pathways. The assays were performed in buffer containing 25 mM Hepes (pH 8.0), 100 mM NaCl, bovine serum albumin (0.1 mg/ml), and 0.01% NP-40. For each experiment, the fluorescently labeled probe was excited at 508 nm, and the emitted light was measured at 528 nm. The fluorescence intensity was recorded in parallel (ΔI∥) and perpendicular (ΔI⊥) planes relative to the polarization of the excitation beam. These intensities were used to calculate the anisotropy (*r*) according to the equation

r=(ΔI∥–ΔI⊥)/(ΔI∥+2ΔI⊥)

Each measurement was performed at room temperature (25°C). Data were normalized using GraphPad Prism, with additional fitting performed using the One Site-Total nonlinear fit equation.

### Separase cleavage assays

^35^S-methionine–labeled human SCC1 was produced in vitro using the TnT coupled wheat extract systems (Promega) according to the manufacturer’s instructions. The system used the TnT T7 Wheat Germ Polymerase, with 0.8 μCi/μl of ^35^S-methionine added to a 50-μl reaction mix, followed by incubation for 1 hour at 30°C. For phosphorylation of SCC1, 4 μM PLK1 was incubated with SCC1 in a buffer containing 25 mM Hepes (pH 7.5), 5 mM ATP, 10 mM MgCl_2_, and 2 mM DTT for 2 hours at 30°C. The cleavage reaction was initiated by mixing 0.25 μM securin^160–202^-separase fusion protein with the SCC1 substrate in a buffer containing 25 mM Hepes (pH 7.7), 5 mM MgCl_2_, 1 mM DTT, and 70 to 90 mM KCl at 30°C. The reaction was terminated by adding 3× SDS-loading buffer. Reaction products were separated by SDS-PAGE using a 4 to 20% gel (Bio-Rad) and visualized with a phosphorimager (Typhoon FLA 9500). Quantification of band intensities was performed using the ImageJ software (*45*).

### Phosphorylation of SCC1-separase fusion complexes

Purified SCC1^100–320^-separase^C2029S/ΔAIL1^ or SCC1^310–550^-separase^C2029S/ΔAIL1^ fusion complex was mixed with the kinase domain of PLK1 at a molar ratio of 1:2 in a reaction buffer containing 50 mM Hepes (pH 8.0), 150 mM NaCl, 10 mM MgCl_2_, 5 mM ATP, 0.5 mM TCEP, and 5% glycerol. The reaction mixture was incubated at room temperature for 1 hour and subsequently applied to gel filtration on a Superose 6 Increase 5/150 column (GE Healthcare Life Sciences) equilibrated with 20 mM Hepes (pH 8.0), 100 mM KCl, and 0.5 mM TCEP. Fractions containing SCC1-separase fusion complexes were directly used for cryo-EM grid preparation.

### MS analysis of phosphorylated SCC1 (amino acids 100 to 550)

Purified SCC1 (amino acids 100 to 550) was incubated with 18 μg of PLK1 for 1 hour at 30°C in a buffer containing 50 mM Hepes (pH 8.0), 150 mM NaCl, 10 mM MgCl_2_, 5 mM ATP, 0.5 mM TCEP, and 5% glycerol. The reaction mixture was subjected to gel filtration to remove PLK1. Peak fractions containing SCC1 (amino acids 100 to 550) were pooled and used for MS analysis of phosphorylation sites.

### Negative staining analysis of cohesin-separase incubated with DNA

DNA sequence from the pericentromeric region of the centromere on human chromosome 18 was cloned into a high-copy plasmid containing two flanking EcoRV restriction sites. Plasmids were amplified in DH5α cells for 16 to 18 hours. The 340–base pair (bp) DNA was purified as previously described (*46*). Final purification of the DNA was performed by anion exchange chromatography. Purified DNA was stored in a buffer containing 20 mM Hepes (pH 8.0), 200 mM KCl, and 1 mM EDTA for further use.

Freshly purified cohesin-separase^C2029S/ΔAIL1^ fusion complex was mixed with 340-bp double-stranded DNA in a molar ratio of 1:1.5 and incubated for 30 min on ice. The sample was then incubated with 1 mM BS3 in a buffer of 20 mM Hepes (pH 8.0), 100 mM KCl, and 0.5 mM TCEP for another 30 min on ice to allow cross-linking. The reaction was terminated by adding 30 mM tris-HCl (pH 8.0). A total of 3 μl of cross-linked sample was applied to carbon-coated 400-mesh copper grids (Electron Microscopy Sciences) that had been glow discharged for 25 s. The sample was incubated on the grid for 1 min before blotting with filter paper to remove excess liquid. Grids were washed with water and stained with 2% (w/v) uranyl formate. A total of 3508 micrographs was acquired on a Talos L120C microscope operated at 120 kV with a nominal magnification of ×57,000, resulting in a pixel size of 2.463 Å. Images were recorded using the EPU software with a defocus range of −1.5 to −2.0 μm. Image processing was performed in CryoSPARC v.4.4.1 (*47*). A total of 302,050 particles of separase bound to SMC subunits was selected and subjected to 3D classification. Three distinct classes showed extra density bound to separase. Full-length human separase and AF3-predicted structures of SMC1-SCC1 and SMC3-SCC1 complexes were fitted into the negative staining EM density maps.

### Cryo-EM sample preparation and data collection

Purified apo-separase (active and inactive) was diluted in a buffer of 20 mM Hepes (pH 8.0), 100 mM KCl, and 0.5 mM TCEP and then applied to graphene oxide–coated holey-carbon grids (Quantifoil R1.2/1.3, 300 mesh, gold) at a concentration of 150 to 200 nM. Graphene oxide grids were prepared following the protocol described in Boland *et al.* (*32*, *48*). The grids were incubated for 10 s and back blotted for 2 to 3 s with 1 mm of additional movement (90% humidity at 16°C) and then plunged into liquid ethane using a Leica EM GP2 automatic plunge freezer. Freshly prepared phosphorylated SCC1-separase fusion complexes were dispensed onto graphene oxide–covered Quantifoil R1.2/1.3 holey-carbon grids at a concentration of 100 to 200 nM. The prepared grids were directly screened and subsequently used for cryo-EM data collection. All datasets were acquired on a Thermo Fisher Scientific Talos Arctica Cryo–transmission EM operating at an accelerating voltage of 200 kV, equipped with either Falcon 3 or Falcon 4i direct electron detector. Data acquisition was monitored on-the-fly preprocessing using CryoSPARC v.4.4.1.

For active apo-separase, a total of 3314 movies and, for inactive apo-separase^C2029S^, a total of 5292 movies were recorded using a Falcon 3 direct electron detector at a nominal magnification of ×150,000, resulting in a pixel size of 0.9759 Å. Data were collected using EPU (Thermo Fisher Scientific) with one image per hole at a defocus range of −0.6 to −2.2 μm and a total electron dose of 40 e^−^/Å^2^ distributed over 40 frames per acquisition.

For phosphorylated SCC1^100–320^-separase^C2029S/ΔAIL1^ or SCC1^310–550^-separase^C2029S/ΔAIL1^ fusion complexes, a total of 12,867 movies or 7451 movies was recorded, respectively, using a Falcon 4i direct electron detector at a nominal magnification of ×130,000, resulting in a pixel size of 0.9024 Å. A postcolumn energy filter (Selectris X) was used for zero-loss filtration with an energy width of 10 eV. Data were collected using EPU (Thermo Fisher Scientific) with one image per hole, a defocus range of −0.6 to −2.0 μm, and a total electron dose of 50 e^−^/Å^2^ distributed over 50 frames per acquisition.

Purified SCC1^310–550^-SA2-separase^C2029S/ΔAIL1^ complex was diluted to a final concentration of either 0.3 mg/ml or 150 to 200 nM in a buffer of 20 mM Hepes (pH 8.0), 150 mM KCl, and 0.5 mM TCEP. A total of 5 μl of diluted sample (at 0.3 mg/ml) was applied onto holey-carbon grids (Quantifoil R1.2/1.3, 300 mesh, gold) and front blotted for 3 to 3.5 s with 1 mm of additional movement (95% humidity at 15°C) before being plunged into liquid ethane using a Leica EM GP2 automatic plunge freezer. Alternatively, 5 μl of diluted sample (at 150 to 200 nM) was applied onto graphene oxide–coated grids, back blotted for 2 to 2.5 s with 1 mm of additional movement (95% humidity at 15°C), and then plunged into liquid ethane using the same plunge freezer. A total of 13,810 movies and 31,617 movies was recorded using a Falcon 4i direct electron detector on standard grids and graphene oxide–coated grids, respectively. Data acquisition was performed at a nominal magnification of ×130,000, resulting in a pixel size of 0.9024 Å, with an energy filter slit width of 10 eV. Each dataset was collected with a total electron dose of 50 e^−^/Å^2^ distributed over 50 frames. Data were collected using EPU (Thermo Fisher Scientific) with three images per hole and a defocus range of −0.6 to −2.0 μm. To attenuate the preferred orientation of particles, an additional 11,585 movies were recorded on graphene oxide–coated grids with a stage tilt of 30° at the same magnification. Data collection for the tilted dataset was performed with a set defocus range of −1.0 to −2.6 μm.

For cryo-EM grid preparation of the cohesin-separase^C2029S/ΔAIL1^-DNA complex, 4 μl of cross-linked sample were applied onto ANTcryo grids (R1.2/1.3, 300 mesh) with an amorphous nickel titanium alloy film. The grids were front blotted for 3 to 3.5 s with 1 mm of additional movement under 95% humidity at 15°C and then plunged into liquid ethane. A total of 22,985 movies was recorded using a Falcon 4i direct electron detector at a nominal magnification of ×130,000, resulting in a pixel size of 0.9024 Å. Data collection was performed using EPU (Thermo Fisher Scientific) with three images per hole, a defocus range of −0.8 to −2.4 μm, and a total electron dose of 50 e^−^/Å^2^ distributed over 50 frames.

### Cryo-EM image processing

All data were processed using CryoSPARC v.4.4.1 (*47*) and RELION 4.0 (*49*). The data processing workflows are summarized in the Supplementary Materials. Raw movies for all datasets were aligned and dose weighed using patch-based motion correction, and contrast transfer function (CTF) parameters were estimated by patch-based CTF estimation in CryoSPARC.

For the wild-type apo-separase dataset, particles were initially picked using a blob picker with a minimum and maximum diameter of 120 and 220 Å. The resulting 2D class averages were used as templates for template-based picking. Particle cleanup using three rounds of 2D classification resulted in 654,549 particles, followed by two rounds of 3D classification. A total of 224,027 particles was selected and subjected to global CTF refinement, local motion correction, and nonuniform refinement, resulting in a map that refined to 3.3-Å resolution. To improve the local density of separase, local refinement was performed using masks focused on the HEAT-repeat domain or the TPR-like and protease domains. A composite map was generated by combining two focused refinement maps.

For the apo-separase^C2029S^ dataset, initial particles were picked by template picker using the templates generated from the active wild-type apo-separase reconstruction. After particle cleanup through three rounds of 2D classification, 852,228 particles were retained for subsequent 3D classification. Resulting 368,728 particles were subjected to global CTF refinement, local motion correction, and nonuniform refinement, resulting in a map at 3.2-Å resolution. Particles were further cleaned up by another round of 2D classification, followed by 3D refinement generating a map at 3.15-Å resolution. To improve the density of separase autocleavage sites, 3D classification without alignment was performed using a mask covering the TPR-like domain and the protease domain. Four classes of particles (118,288 particles) showing improved density of autocleavage sites were selected for 3D refinement, yielding a map at 3.1-Å resolution. Local resolution of the TPR-like domain and the protease domain was further improved by local refinement.

For the phosphorylated SCC1^100–320^-separase^C2029S/ΔAIL1^ dataset, templates generated from apo-separase^C2029S^ reconstruction were used and subsequently cleaned up by four rounds of 2D classification and two rounds of 3D classification. A total of 518,130 particles was used for nonuniform refinement and CTF refinement and resulted in a reconstruction of 2.9-Å resolution. To improve the occupancy of SCC1, refined particles were imported into RELION for 3D classification without alignment (*K* = 5 and *T* = 8), using a mask on SCC1. The class (214,707 particles) showing the strongest SCC1 density was selected, and particles were subjected to CTF refinement and nonuniform refinement. Another round of 3D classification without alignment (*K* = 6 and *T* = 12) was performed in RELION, using a mask covering the TPR-like and protease domains and SCC1. The best class showing high-resolution features was selected (195,231 particles) for 3D refinement in CryoSPARC, resulting in a map at 2.9-Å resolution. Local refinement was performed to further improve the density of SCC1.

For the phosphorylated SCC1^310–550^-separase^C2029S/ΔAIL1^ complex, the processing strategies were similar to those described above. Briefly, after data cleanup through 2D and 3D classifications, 298,574 particles were retained for subsequent CTF refinement and nonuniform refinement, followed by local refinement focused on the TPR-like and protease domains. This resulted in a reconstruction at a resolution of 2.9 Å. 3D classification without alignment (*K* = 6 and *T* = 8) in RELION using a mask on the HEAT-repeat domain of separase resulted in a class (180,532 particles) with clear density of SCC1 binding to separase. The particles were reimported into CryoSPARC for nonuniform refinement, followed by a final local refinement with a soft mask around the HEAT-repeat domain. As a result, the density of SCC1 binding to the HEAT-repeat domain was notably improved.

For the SCC1^310–550^-SA2-separase^C2029S/ΔAIL1^ complex, untilted and tilted (30°) datasets were processed separately until the 2D classification step. Initial particles were picked by a template picker combined with a blob picker and cleaned up through several rounds of 2D classification. Particles corresponding to the tertiary complex, SCC1^310–550^-separase and SCC1-SA2 complexes, from each dataset were selected and merged for further processing. A total of 795,457 particles for the tertiary complex was subjected to four rounds of 3D classification. The resulting 382,898 particles were used for CTF refinement and nonuniform refinement, followed by further data cleanup through 2D and 3D classification. Particles of the best class and particles corresponding to the tertiary complex from other classes were selected (total of 177,878 particles). 3D refinement using nonuniform refinement generated a reconstruction at 3.4-Å resolution. Local refinement using a soft mask 1 covering SCC1, separase, and C-terminal helices of SA2 notably improved the density of the interaction interface and reduced the anisotropy of 3D reconstruction, producing a map at 3.1-Å resolution. A soft mask 2 on SA2, SCC1, and part of the protease domain was also applied to local refinement improving the map of SCC1-SA2. A composite map of the tertiary complex was created by combining two focused-refined maps. 3D classification of 1,375,401 particles for SCC1^310–550^-separase complex resulted in a best class with 614,186 particles. Following CTF refinement and nonuniform refinement yielded a map at 2.9 Å-resolution. Local refinement using soft masks on each domain further improved the local densities. The focused-refined maps were used to generate a composite map of SCC1^310–550^-separase complex. Similarly, after 3D classification of a total of 2,823,666 particles for SCC1^310–550^-SA2 complex, the best class containing 768,540 particles was selected for further CTF refinement and nonuniform refinement. Subsequent local refinement was performed using soft masks covering the N-terminal part (mask 3) or C-terminal part (mask 4) of SA2. The two locally improved maps were combined to produce a composite map.

For the cohesin-separase ^C2029S/ΔAIL1^-DNA complex dataset, particles were initially picked using both blob template pickers. Following several rounds of 2D classification and removal of duplicated particles, the resulting 2D class averages showing separase bound to cohesin subunits were used as input for the Topaz particle-picking pipeline to increase the number and accuracy of picked particles. After cleanup through multiple rounds of 2D and 3D classifications, 179,047 particles were subjected to CTF refinement and nonuniform refinement. The resulting map was refined to an overall resolution of 3.4 Å. To improve the density of cohesin subunits bound to separase, 3D classification without alignment (six classes) was performed in CryoSPARC. One class (26,747 particles) showing enhanced density of cohesin subunits was selected for a final nonuniform refinement, producing a reconstruction of 4.3 Å.

All maps were postprocessed with a 3D deep learning framework named EMReady (*50*). All resolution estimations were derived from Fourier shell correlation (FSC) calculations between reconstructions from two independently refined half-maps. Reported resolutions are based on the FSC gold standard criterion. Local resolution estimations are obtained by ResMap (*51*).

### Model building and refinement

The cryo-EM structure of human separase-securin complex (7NJ1) was used as an initial reference to model the structures of apo-separase (active and inactive), SCC1^100–320^-separase^C2029S/ΔAIL1^, and SCC1^310–550^-separase^C2029S/ΔAIL1^ fusion complexes. Model building of SCC1 in regions that were difficult to interpret was facilitated by automated atomic model building using ModelAngelo (*52*) and AlphaFold predictions (*40*). Similarly, the crystal structure of SCC1-SA2 [4PJU (*41*)] together with the SCC1^310–550^-separase^C2029S/ΔAIL1^ structure from this study were used as initial models for building the structure of the SCC1^310–550^-SA2-separase^C2029S/ΔAIL1^ complex. All initial models were fitted into cryo-EM maps using ChimeraX (*53*), manually built in Coot (*54*), and real-space refined using PHENIX (*55*). Model validation was performed in MolProbity (*56*). Structural figures were generated in ChimeraX. All statistics are summarized in table S1, and a summary of all structural models, with PDB ID, is provided in table S2.

### Sample preparation for XL-MS

The purified cohesin-separase^C2029S/ΔAIL1^ fusion complex was mixed with 340-bp double-stranded DNA in a molar ratio of 1:1.2 and incubated for 30 min on ice. The sample was then incubated with 0.25 mM Sulfo-SDA (Thermo Fisher Scientific Pierce) in a buffer of 20 mM Hepes (pH 8.0), 100 mM KCl, and 0.5 mM TCEP for 10 min at room temperature. The mixture was transferred onto Eppendorf tube lids, placed on ice, and irradiated with UV light at 365 nm for 10 s using an LED Spot 100 HP IC (Honle UV Technology). Reactions were quenched with 50 mM ammonium bicarbonate for 10 min and then precipitated by adding four volumes of −20°C cold acetone and incubating overnight at −20°C. Precipitated material was pelleted by centrifugation at 20,000*g* for 15 min. The pellet was briefly washed in cold acetone, pelleted again, and air dried. The cross-linked samples were resuspended in 8 M urea and 100 mM ammonium bicarbonate. Proteins were reduced with 5 mM DTT, alkylated with 10 mM iodoacetamide, and digested with lysyl endopeptidase at an enzyme-to-protein ratio of 1:100 for 4 hours at room temperature with shaking. Urea was diluted to 1.5 M with 100 mM ammonium bicarbonate solution, and the peptides were further digested with trypsin at an enzyme-to-protein ratio of 1:50 overnight at room temperature with shaking. Digested peptides were cleaned and desalted by C18 StageTips and were dried in a vacuum concentrator (Eppendorf). For cross-linked peptide enrichment, peptides were fractionated on an ÄKTA Pure system (GE HealthCare) using a Superdex 30 Increase 3.2/300 (GE HealthCare) at a flow rate of 10 μl/min using 30% (v/v) acetonitrile (ACN) and 0.1% (v/v) trifluoroacetic acid as the mobile phase at 4°C. A total of 50 μl fractions was collected from the elution volume of 1.00 to 1.45 ml and dried for subsequent liquid chromatography–tandem MS (LC–MS/MS) analysis.

### Liquid chromatography and XL-MS

All peptides were resuspended in 0.1% formic acid (FA) and 1.6% ACN before being analyzed on a Thermo Fisher Scientific Eclipse Orbitrap MS coupled to a Vanquish Neo HPLC using an EASY-Spray source and PepMap Neo 75 μm × 500 mm C18 column at 0.3 μl/min. A 95-min gradient was used (1.6 to 44% ACN/0.1% FA), followed by 44 to 76% ACN/0.1% FA for 2.5 min. Global MS parameters included a 95-min total method, advanced peak determination checked default charge state of 2, EASY-IC internal calibration, static spray voltage of 2 kV, sweep gas of 2, ion transfer tube (ITT) temperature of 280°C, expected liquid chromatography (LC) peak widths of ~30 s, and positive polarity. Fractions 1 to 9 were analyzed with different acquisition methods (frac1: A, frac2: A/B/E, frac3: A/B/C/E, and frac4 to 9: A/D/E). Acquisition A (duty cycle: 3 s) used an Orbitrap MS1 at 240,000 resolution [mass/charge ratio (*m/z*): 380 to 2000], quadrupole isolation, automatic gain control (AGC) target of 150% (max injection time: 100 ms), one microscan in profile mode, and dynamic exclusion [±10 parts per million (ppm) for 10 s] for charge states 3 to 7, with subbranches defining precursor selection ranges for 4+ (*m/z*: 380 to 1800), 5+ (*m/z*: 380 to 1350), and 6 and 7+ (*m/z* 380 to 1000). ddMS2 scans were Orbitrap HCD at 60,000 resolution (*m/z*: 150 to 2000), isolation window of 1.4 *m/z*, stepped collision energies of 20, 26, and 30 (normalized), an AGC target of 750% (max injection time: 150 ms), one microscan in centroid mode, and charge 3+ as second priority. Acquisition B, C, and D followed the same setup but with targeted precursor selection ranges: B (*m/z*: 750 to 1005), C (*m/z*: 1000 to 1800), and D with three windows (*m/z*: 380 to 655, 650 to 905, and 900 to 1800, respectively). Acquisition E (“Boxcar”) used a 5.12-s duty cycle with targeted selected ion monitoring (SIM) at 240,000 resolution, multiplex isolation of 10 ions (user-defined groups), custom AGC target with autoinjection time, and source fragmentation at 10 V; two boxcar mass lists specified the center *m/z* values, isolation windows (Box1: 414.1/30.2, 462.2/24.8, 504.1/23.6, 545.35/23.1, 587.8/25, 634.05/26.7, 686/31.4, 784.45/38.9, 829.65/48.5, 957.2/91.6; Box2: 439.5/26.6, 483.45/23.7, 524.85/23.9, 566.15/24.3, 610.5/26.4, 658.85/28.9, 715.35/33.3, 786.65/43.5, 882.65/63.5, 1101/202), and an AGC target of 50%. All other parameters were consistent with acquisitions A to D.

### Data analysis of XL-MS

A recalibration to control for detector error was conducted on MS1 and MS2 based on the median mass shift of high-confidence [<1% false discovery rate (FDR)] linear peptide identifications from each raw file. To identify cross-linked peptides, the recalibrated peak lists were searched against the sequences and the reversed sequences (as decoys) of cross-linked peptides using the Xi software suite (v.1.8.6; https://github.com/Rappsilber-Laboratory/XiSearch). The following parameters were applied for the search: MS1 accuracy = 2 ppm; MS2 accuracy = 5 ppm; enzyme = trypsin allowing up to three missed cleavages and two missing monoisotopic peaks; cross-linker = SDA with an assumed *N*-hydroxysuccinimide–ester reaction specificity for K, Y, S, T, and N termini; diazirine reaction specificity for A, Ccm, D, E, G, H, I, K, L, P, S, T, V, Y, C termini, and N termini; fixed modifications = carbamidomethylation on cysteine; variable modifications = acetylation on lysine and protein N termini, oxidation on methionine, hydrolyzed SDA on lysines, and protein N termini. MS cleavage of SDA cross-links was considered during searches.

Before estimating the FDR, the matches were filtered to those having greater than two fragments matched with a noncleaved SDA and at least five matches total per peptide. These candidates were then filtered to a 1% FDR at the residue-pair level using xiFDR (v.2.3.2) (*57*).
