# Supplementary material for: Substrate recognition by human separase
Source: Sci Adv. 2025 Nov 12;11(46):eady9807. doi: 10.1126/sciadv.ady9807 (PMC12609080; doi:10.1126/sciadv.ady9807)
Supplement: Supplementary file 1 — Figs. S1 to S15 Tables S1 to S3 Legend for data S1 Legend for movies S1 and S2 [file sciadv.ady9807_sm.pdf]

Science Advances
AAAS

Supplementary Materials for

Substrate recognition by human separase

Jun Yu *et al.*

Corresponding author: Andreas Boland, Andreas.Boland@unige.ch; Jun Yu, jun.yu@unige.ch

Sci. Adv. **11**, eady9807 (2025)
DOI: 10.1126/sciadv.ady9807

The PDF file includes:

Figs. S1 to S15
Tables S1 to S3
Legend for data S1
Legends for movies S1 and S2

Other Supplementary Material for this manuscript includes the following:

Data S1
Movies S1 and S2

Figures S1 to S15

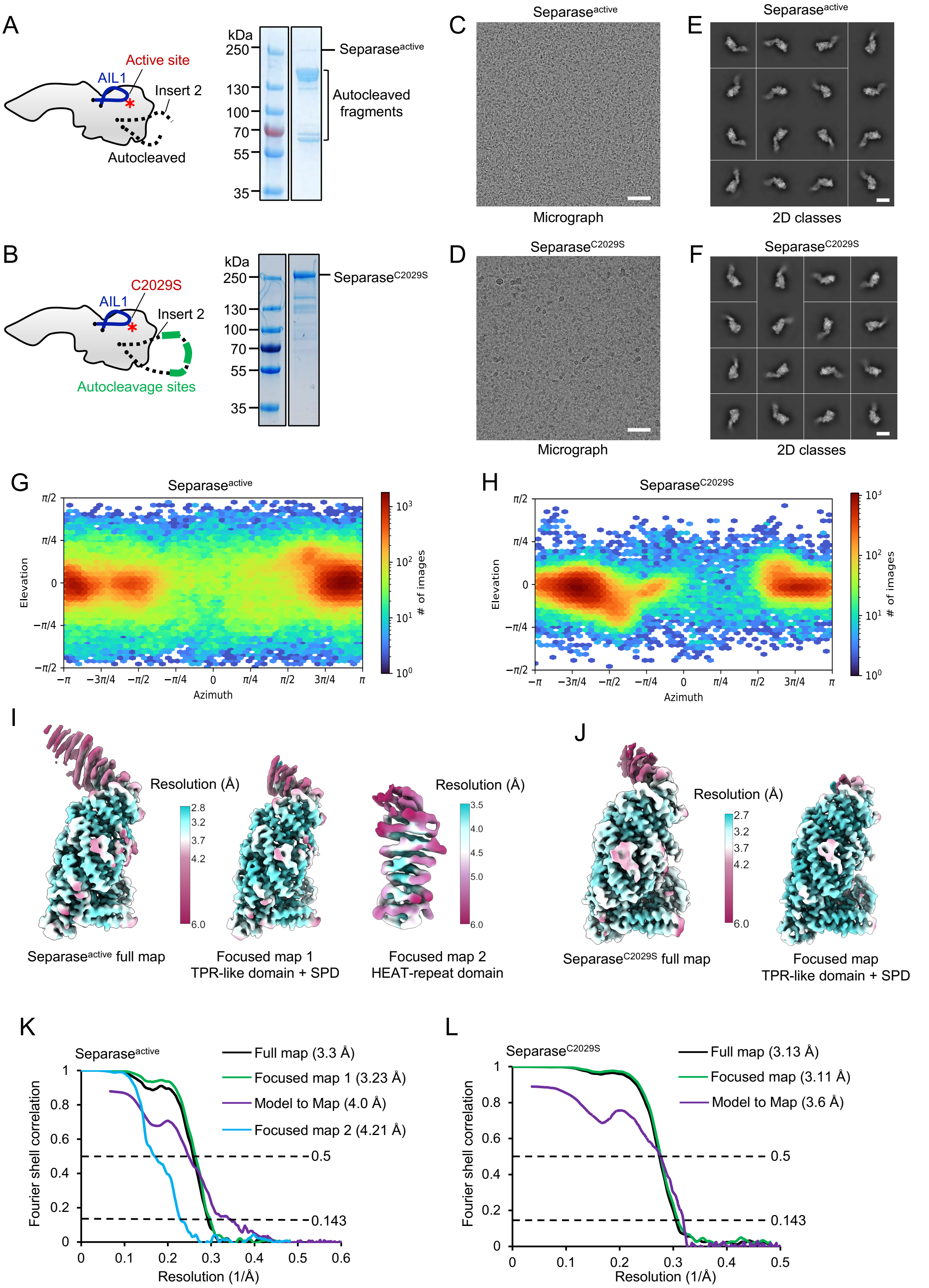

Figure S1. Biochemical and cryoEM analysis of active and inactive apo-separase. A, SDS-PAGE gel of active separase, showing that separase undergoes autocleavage during expression and

purification. A schematic cartoon that indicates AIL1, cleaved insert 2 and the active site is shown on the left. **B,** SDS-PAGE gel of inactive separase. C2029S mutation prevents separase autocleavage**.** A schematic cartoon that indicates AIL1, intact insert 2 and the active site is shown on the left. **C-D,** Representative micrographs of active separase (**C**) and inactive separase (**D**), collected on graphene oxide-coated grids to increase the number of views of separase (*32*, *48*). Scale bars, 500 Å. **E-F,** Gallery of two-dimensional class averages of active (**E**) and inactive (**F**) separase, showing typical classes of various views. Scale bars, 100 Å. **G-H,** Angular distribution of active (**G**) and inactive (**H**) separase. Data sets calculated using non-uniform refinement algorithm in CryoSPARC (*47*). **I,** EM density maps of active separase colour-coded according to local resolution ranging from 2.8 Å to 6 Å for full map and focussed refined map 1 (TPR-like and protease domains), and 3.5 Å to 6 Å for focussed refined map 1 (HEAT-repeat domain) (*51*). **J,** EM density maps of inactive separase colour-coded according to local resolution ranging from 2.8 Å to 6 Å for full map and focussed refined map (TPR-like and protease domains). **K-L,** Gold standard Fourier Shell Correlation (FSC) curves of the full map and the focused refined maps for active separase (**K**) and inactive separase (**L**). The FSC curve between the full cryoEM map and the final atomic coordinates is calculated using Mtriage (*58*).

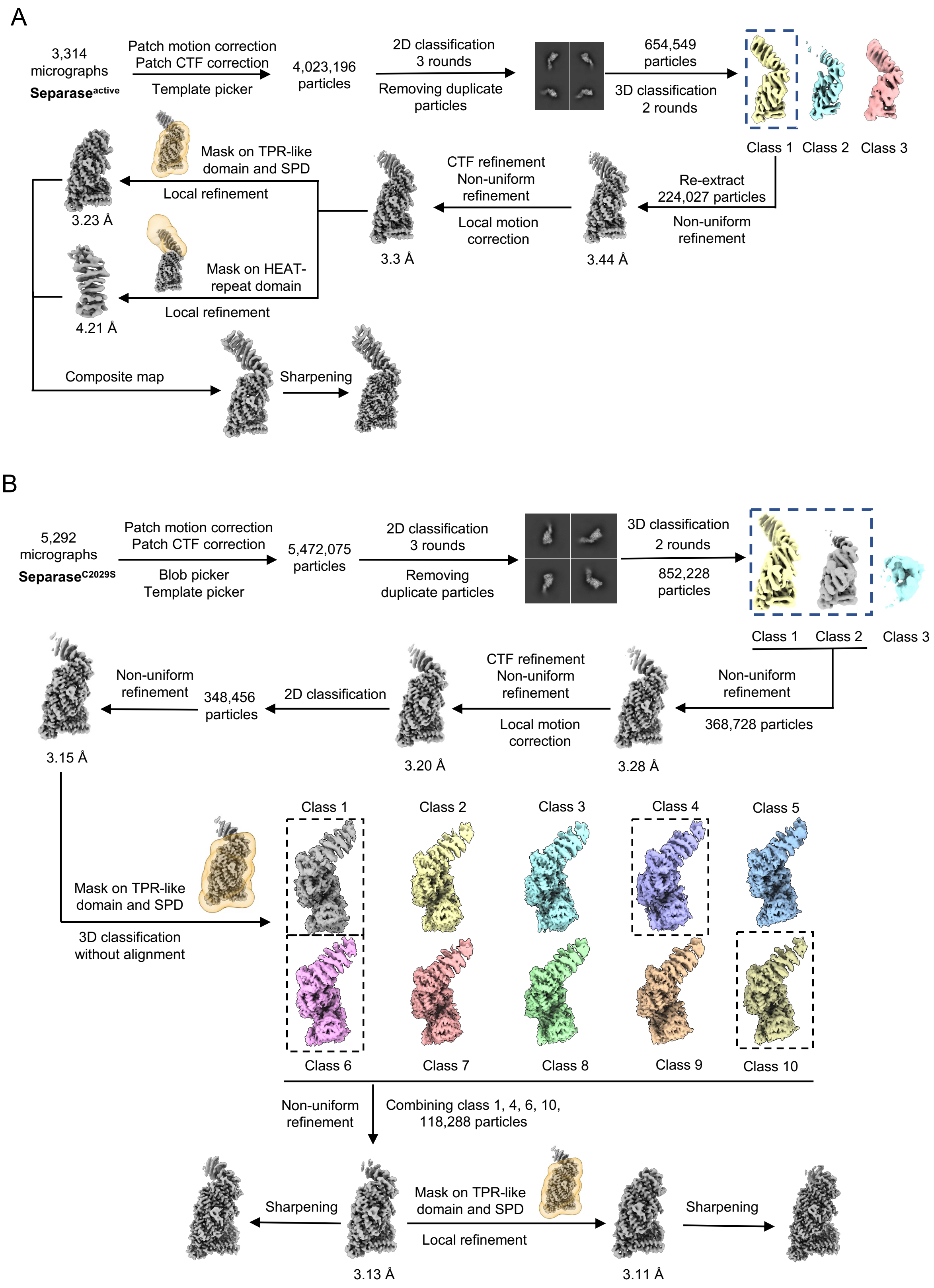

Figure S2. Data-processing flowcharts for both active and inactive apo-separase. A, CryoEM processing pipeline of active separase. Initial particles picked from approximately 3,300 micrographs were cleaned up by three rounds of 2D classification and two rounds of 3D classification. The final set of 224,027 particles were subjected to CTF refinement, local motion correction and non-uniform

refinement, resulting in a map at 3.3 Å resolution. Local refinement was performed using masks on the TPR-like and protease domains (top), and the HEAT-repeat domain of separase (bottom), yielding maps at 3.2 Å and 4.2 Å, respectively. A composite map was generated by combining the two focussed refined maps. **B,** CryoEM processing pipeline of inactive separase. Following initial cleanup through 2D and 3D classifications, 348,456 particles were selected. These particles were further subjected to non-uniform refinement and 3D classification without alignment, applying a mask on the TPR-like and protease domains. Four classes of particles showing clear density for autocleavage fragment were combined and refined, resulting in a final map at 3.1 Å resolution. A final local refinement using a mask on the TPR-like and protease domains was performed. This map also refined to 3.1 Å resolution.

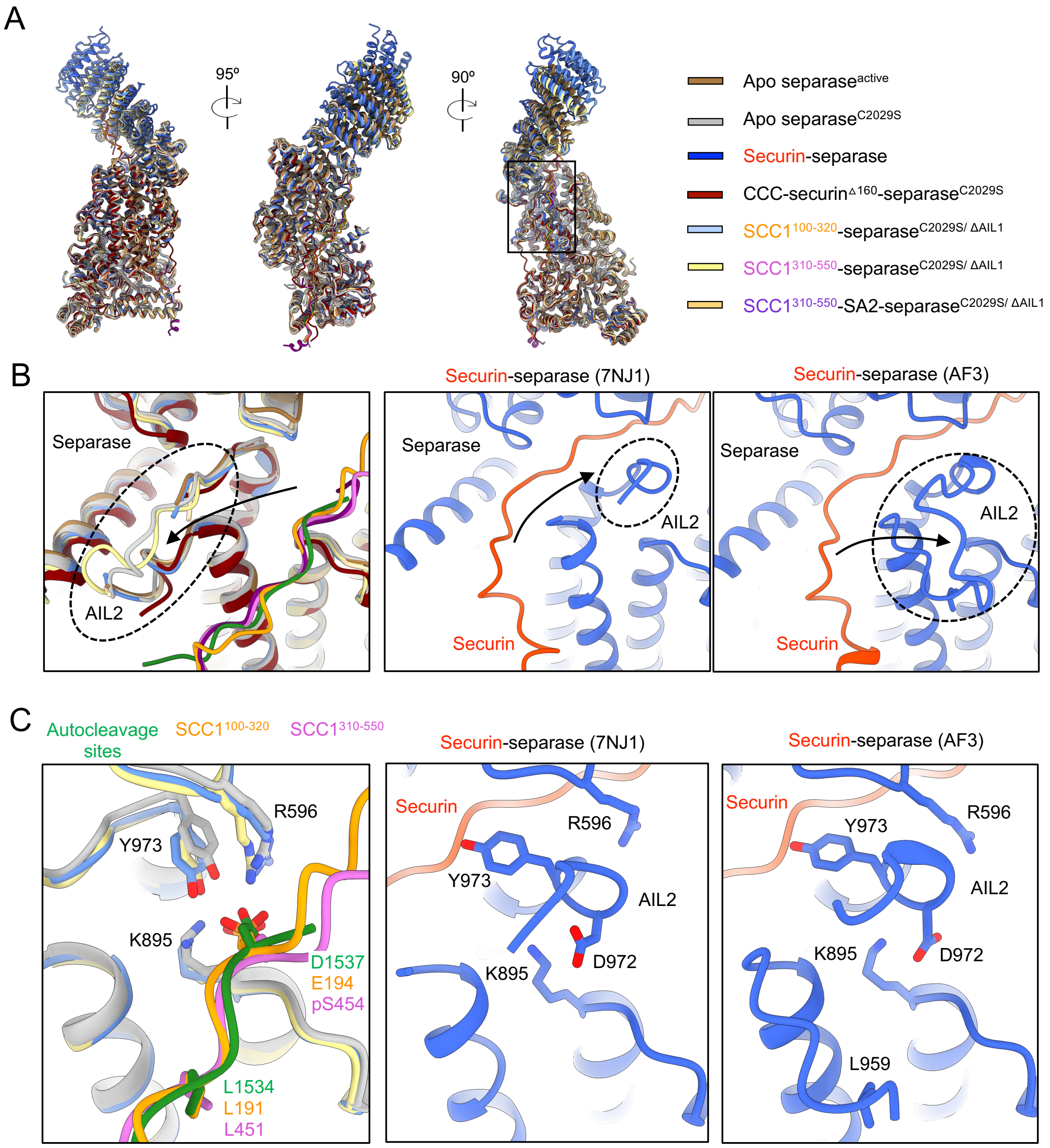

Figure S3. Local conformational changes of separase structures in apo, substrate-bound, and inhibitor-bound states A, Structural alignment of separase bound to substrates, inhibitors and in apo states. Structures are shown as cartoons with separase coloured by different blocks. Securin is in orange red; autocleavage sites are in forest green; SCC1 (aa 100-320) is in orange; SCC1 (aa 310-550) is coloured orchid in the binary complex and purple in the tertiary complex (with SA2). **B,** Conformational change of AIL2. In apo-separase, SCC1-separase and CCC-separase complexes, AIL2 (circled by a dashed line, left panel) binds to a hydrophobic groove on separase. In the securin-separase complex (middle panel), securin occupies the hydrophobic groove while AIL2 binds at the substate-binding site. Alphafold-predicted structure of securin-separase complex reveals the complete AIL2 structure (right panel). The black arrows indicate the movement of AIL2 between complexes. **C,** Close-up view showing AIL2 occupies Lxx[S/D/E] motif-binding site in the securin-separase complex. Left,

Lxx[S/D/E] motif from the autocleavage sites and SCC1 site 1 and site 2 binds to the TPR-like domain of separase. Middle and right, residues L959 and D972 of AIL2 occupy Lxx[S/D/E] motif-binding site.

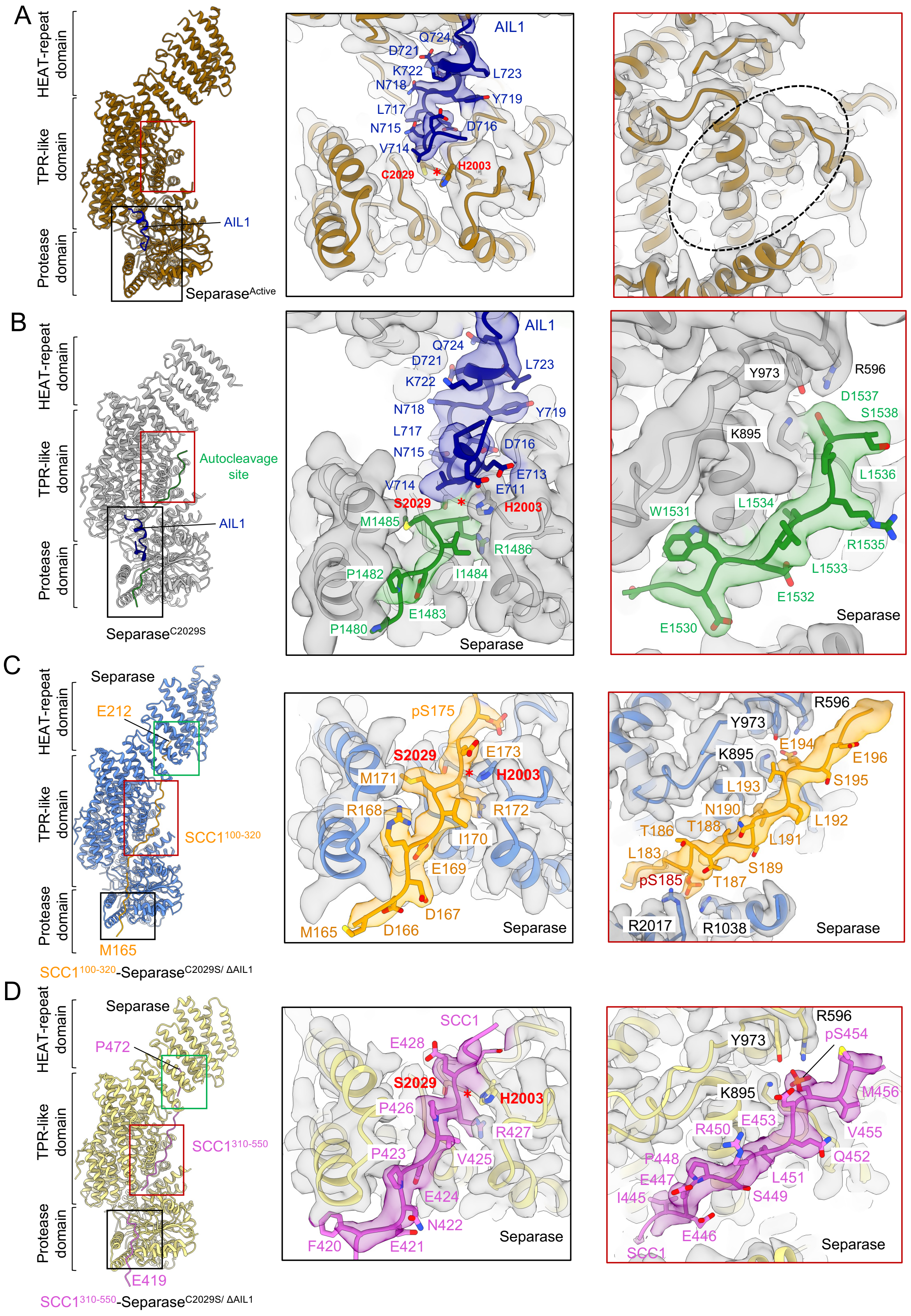
A
HEAT-repeat domain
TPR-like domain
Protease domain
AIL1
Separase^Active
Q724
D721
K722
N718
L717
N715
V714
L723
Y719
D716
C2029
H2003
B
Autocleavage site
Separase^C2029S
E713
E711
S2029
M1485
R1486
P1482
I1484
E1483
P1480
Separase
Y973
R596
D1537
S1538
K895
L1536
W1531
L1534
R1535
L1533
E1532
E1530
C
E212
SCC1^100-320
M165
SCC1^100-320-Separase^C2029S/ ΔAIL1
pS175
E173
M171
R168
R172
I170
E169
D167
D166
E194
E196
S195
L193
N190
L192
T186
T188
L191
L183
S189
pS185
T187
R2017
R1038
D
P472
SCC1^310-550
E419
SCC1^310-550-Separase^C2029S/ ΔAIL1
SCC1
E428
P426
R427
P423
V425
E424
N422
F420
E421
pS454
M456
E453
R450
V455
P448
Q452
E447
L451
I445
S449
E446

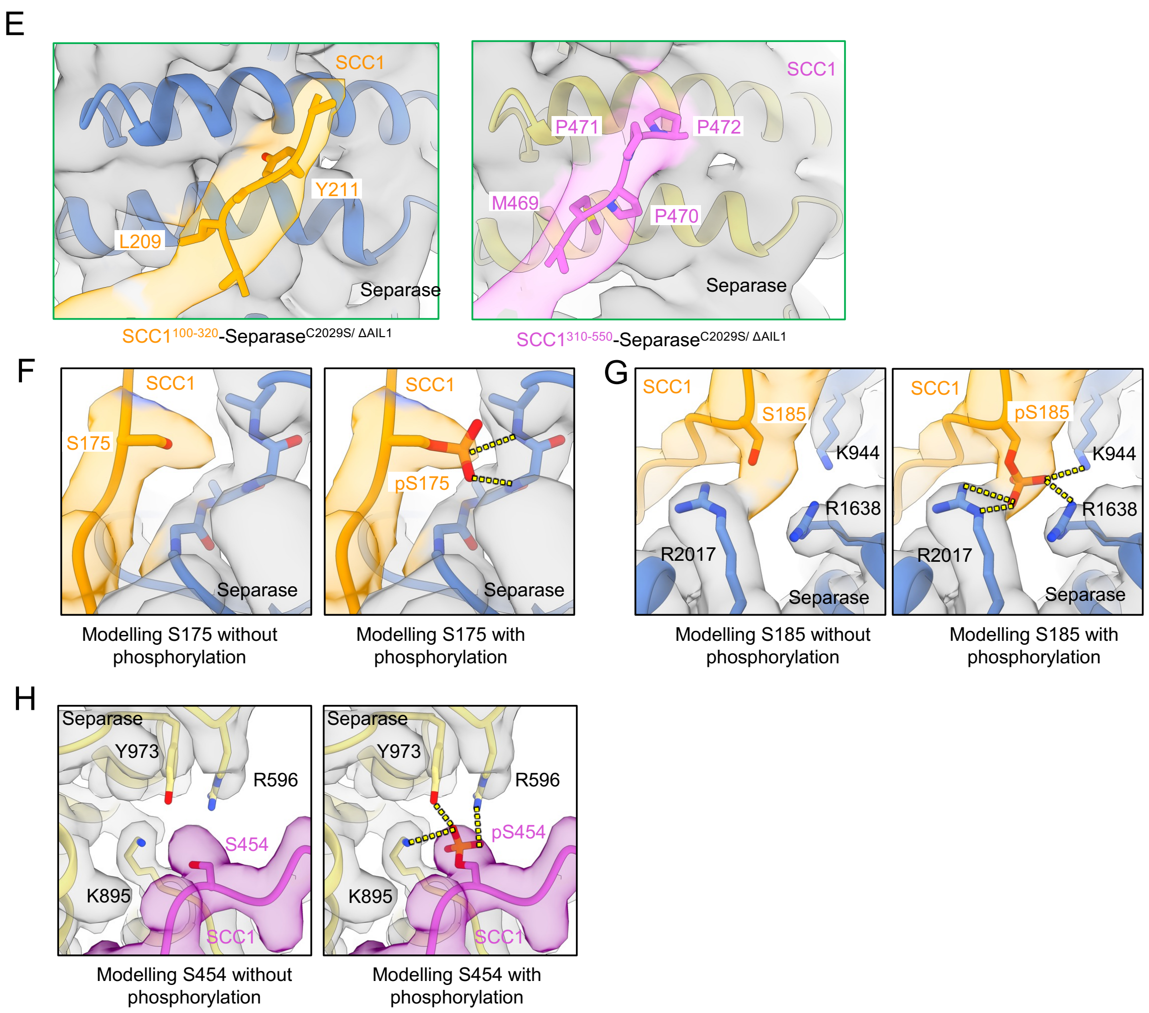

Figure S4. Representative EM density of separase in apo or substrate-bound states. A, Ribbon representation of active apo-separase on the left with AIL1 in blue. EM density of AIL1 binding near the catalytic site (black box) and the TPR-like domain of active separase (red box). The dashed-line circle indicates no extra density binding in this region. **B,** Ribbon representation of inactive apo-separase on the left with AIL1 in blue and autocleavage sites in green. EM density of the autocleavage site 1 and AIL1 binding to the protease domain (black box), and the autocleavage site 3 binding to the TPR-like domain of inactive separase (red box). **C-D,** Ribbon representation of inactive separase bound to site 1 (**C**) or site 2 (**D**) on the left. EM density of SCC1 (aa 100-320) (**B**) and SCC1 (aa 310-550) (**C**) bound to separase. Black box, density showing the cleavage sites 1 and 2 motifs binding to the protease domain. Red box, density of exosites in SCC1 binding to the TPR-like domain. **E,** Close-up views (green boxes in **B** and **C**) of EM density showing SCC1 site 1 (left) and site 2 (right) bound to the HEAT-repeat domain of separase. **F-H,** EM densities of SCC1 serine residues modelled with and without phosphate group. All densities clearly indicate that S175, S185 and S454 of SCC1 are phosphorylated.

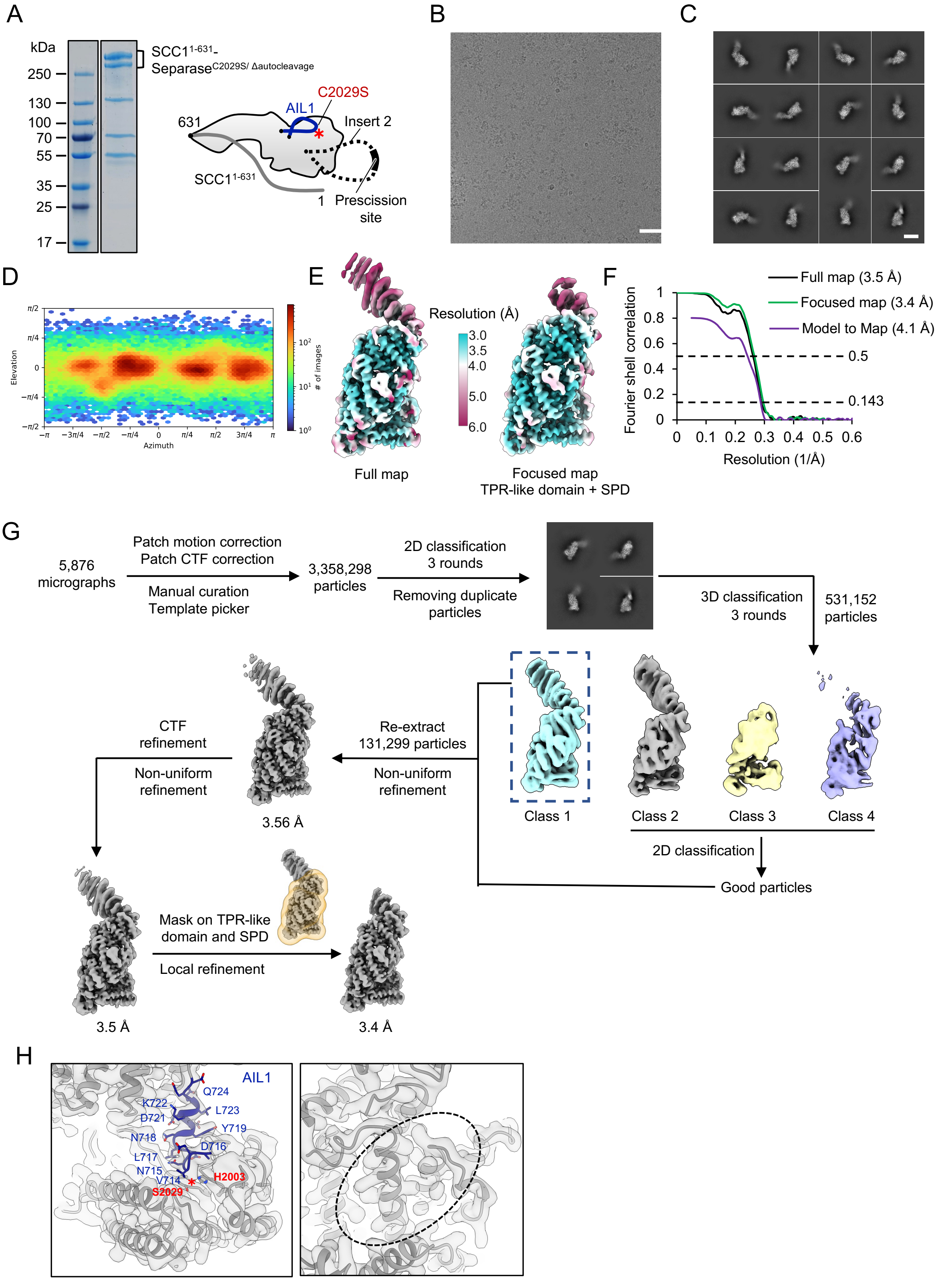

Figure S5. Biochemical and cryoEM analysis of SCC1$^{1\text{-}631}$-separase$^{C2029S/\Delta autocleavage}$ fusion complex. **A,** Schematic representation and SDS-PAGE gel of the fusion complex. In this construct, the C-terminus of SCC1 was fused to the N-terminus of separase with a GS linker in between and the autocleavage sites (aa 1482-1536) within insert 2 was replaced by a preScission site (Δautocleavage). **B,**

Representative cryo-electron micrographs of the fusion complex, collected on graphene oxide-coated EM grids to increase the number of views of separase. Scale bars, 500 Å. **C,** Representative two-dimensional class averages of the fusion complex. Scale bars, 100 Å. **D,** Angular distribution plot for the fusion complex data set calculated using non-uniform refinement algorithm in CryoSPARC (*47*). **E,** EM density maps of the fusion complex colour-coded according to local resolution. **F,** Gold standard FSC curves of the full map and the focused refined maps. The FSC curve between the full cryoEM map and the final atomic coordinates is calculated using Mtriage (*58*). **G,** CryoEM processing pipeline of the fusion complex. The final refinement includes 131,299 particles and the maps refines to a resolution of 3.4 Å. **H,** Representative EM density of AIL1 binding near the catalytic site (left) and the TPR-like domain of separase (right) in the fusion complex. AIL1 is shown in dark blue, and the EM density is shown in light grey. The dashed-line circle indicates no extra density binding in the TPR region as observed for SCC1 site 1 and site 2 or the autocleavage site 3.

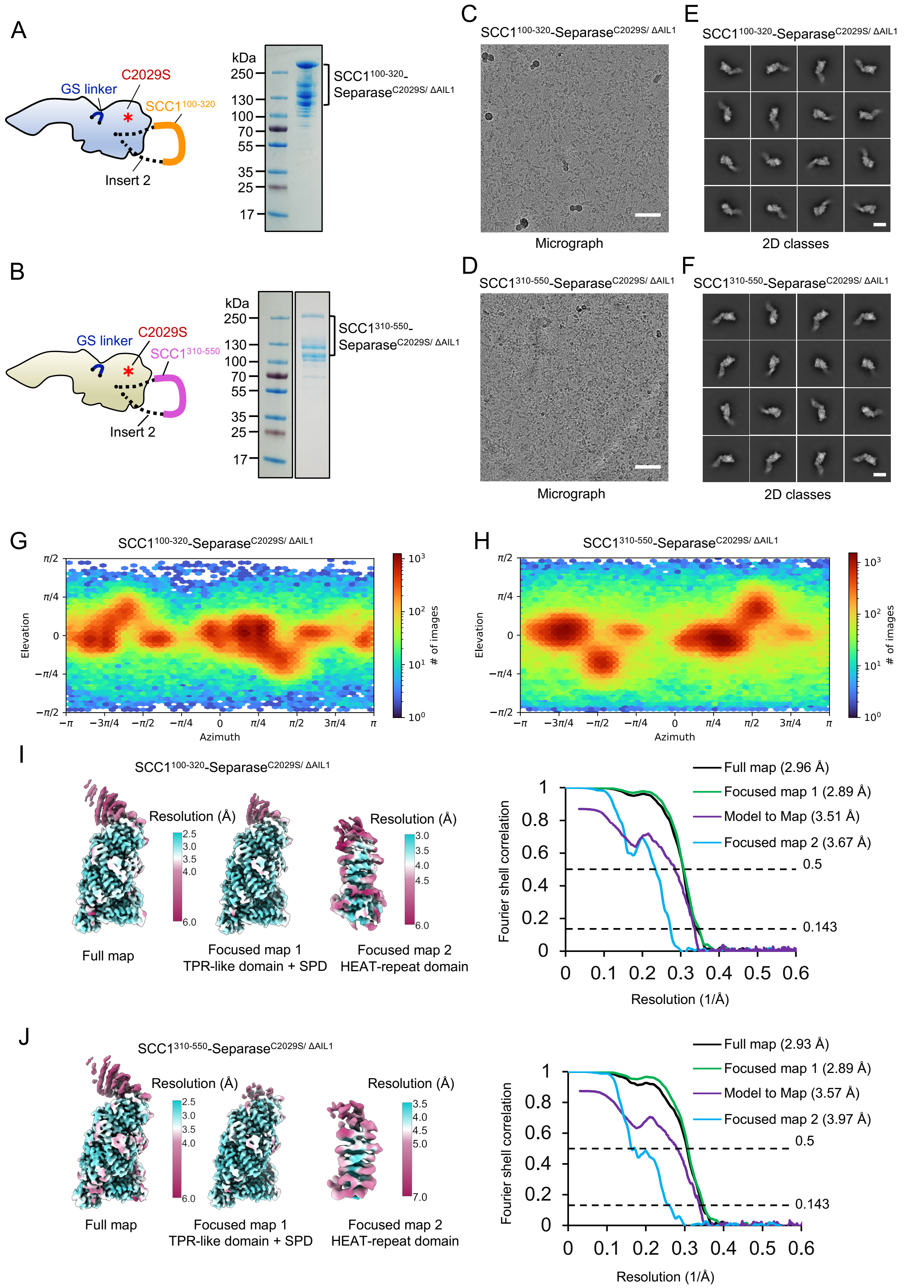

Figure S6. Biochemical and cryoEM analysis of separase bound to SCC1$^{100\text{-}320}$ or SCC1$^{310\text{-}550}$. A-B, SDS-PAGE gels of SCC1$^{100\text{-}320}$-separase$^{C2029S/\Delta AIL1}$ complex (**A**) and SCC1$^{310\text{-}550}$-separase$^{C2029S/\Delta AIL1}$

complex (**B**). Here, SCC1 fragments replaced the autocleavage sites (aa 1482-1536) in insert 2 of separase. ∆AIL1, deletion of AIL1. **C-D,** Representative EM micrographs of the two complexes, collected on graphene oxide-coated grids to increase particle orientation and distribution. Scale bars, 500 Å. **E-F,** Gallery of two-dimensional class averages of the two complexes, showing typical classes of various views. Scale bars, 100 Å. **G-H,** Angular distribution plots for the two complexes calculated using non-uniform refinement algorithm in CryoSPARC (*47*). **I-J,** EM density maps of the two complexes colour-coded according to local resolution and gold standard FSC curves for the full map and the focussed refined maps. The FSC curves between the full cryoEM map and the final atomic coordinates were calculated using Mtriage.

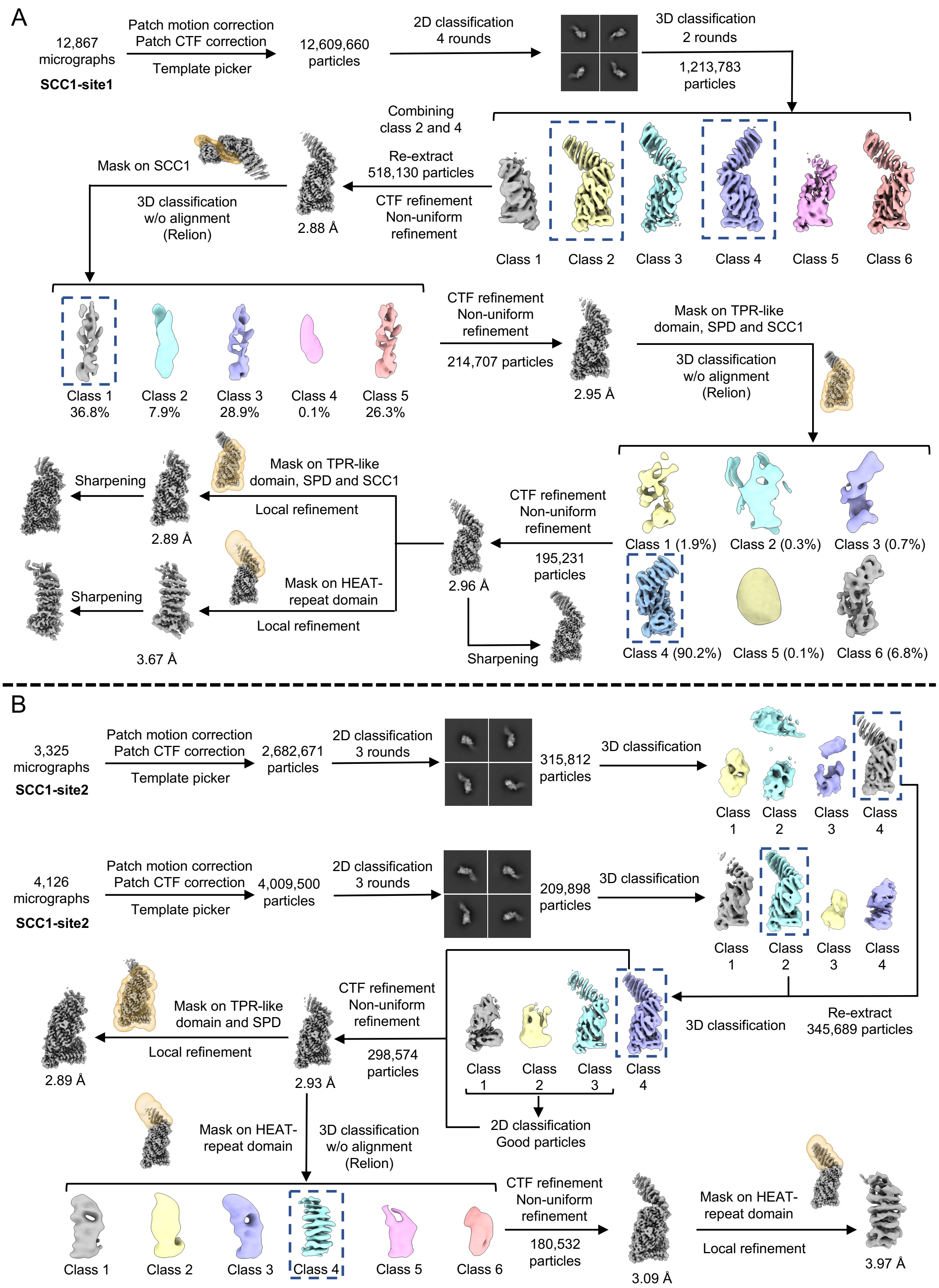

Figure S7. Data-processing flowcharts for separase bound to SCC1$^{100\text{-}320}$ or SCC1$^{310\text{-}550}$. A, CryoEM processing pipeline of SCC1$^{100\text{-}320}$-separase$^{C2029S/\Delta AIL1}$ complex. After four rounds of 2D classification and two rounds of 3D classification, 518,130 particles were selected and subjected to CTF refinement and non-uniform refinement. 3D classification without alignment was performed to further

improve SCC1 density using masks focussed on SCC1 alone and subsequently on SCC1 combined with the C-terminal domains of separase (TPR-like and protease domains). Local refinement with a mask on the HEAT-repeat domain of separase produced a map at 3.67 Å resolution (bottom), improving the density of SCC1 binding to the N-terminal HEAT-repeat domain. **B,** CryoEM processing pipeline of $SCC1^{310-550}$-$separase^{C2029S/\Delta AIL1}$ complex. Two data sets (3,325 and 4,126 micrographs) were combined. Following 3D classification 298,574 particles were selected and subjected to CTF refinement, non-uniform refinement, and local refinement. The EM density of the HEAT-repeat domain of separase was improved by 3D classification without alignment and local refinement using a mask covering this domain.

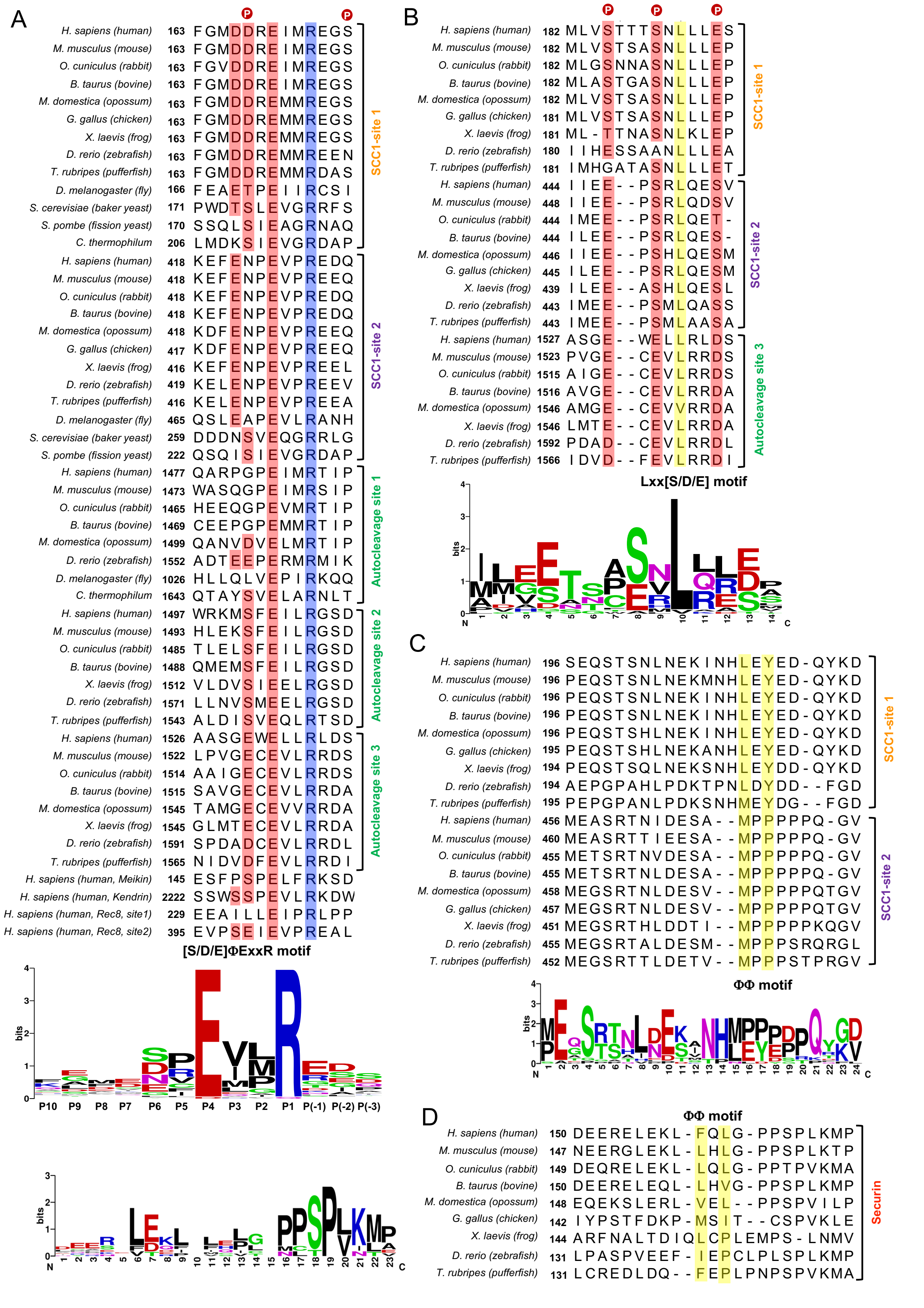

Figure S8. Sequence alignment of substrate motifs. A, Sequence alignment of [S/D/E]φExxR motif in SCC1 and autocleavage sites of separase from various species. Residues binding to P-sites 1-5 are

2 labelled as white Ps in red circles. **B,** Sequence alignment of Lxx[S/D/E] motif in SCC1 and autocleavage site 3 of separase. **C-D,** Sequence alignment of φφ motif in SCC1 and securin. Sequence logos were generated using WebLogo (*59*).

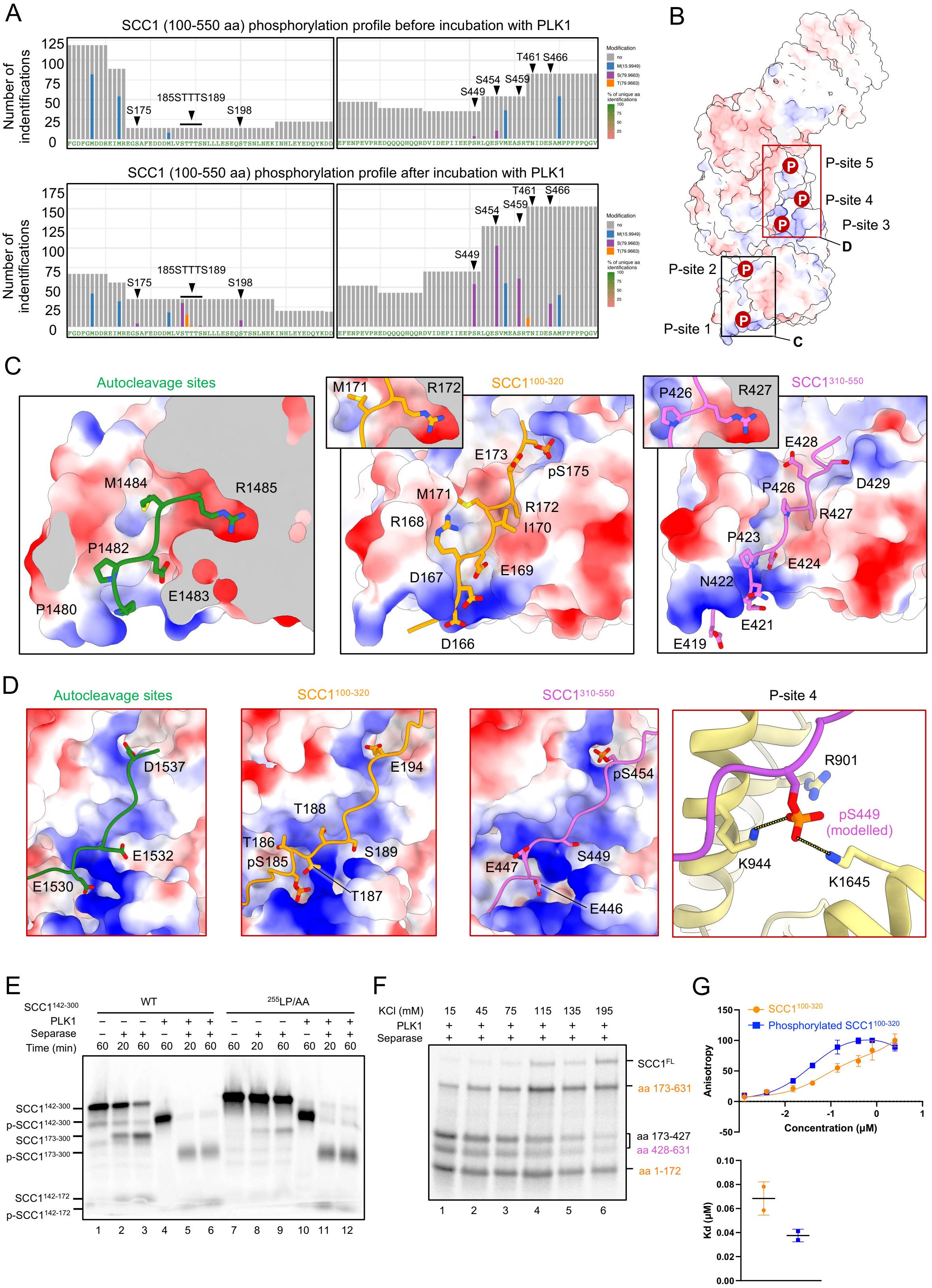

Figure S9. SCC1 binding to separase is primarily mediated by electrostatic interactions. A, Mass spectrometry analysis of SCC1 (aa 100-550) after incubation (bottom) with 5 mM ATP, 10 mM Mg^{2+} and 18 µg PLK1, showing the (partial) phosphorylation of residues S175, S185, T186, T187, S189,

S449 and S454. **B,** Electrostatic surface potential of the interaction interface between substrates and separase. Five phosphate-binding sites on separase are indicated as white Ps in red circles. Close-up views of substrate binding sites are highlighted with black boxes. **C,** Close-up view (black box in **B**) showing the autocleavage site 1 and cleavage site motifs binding to the protease domain. The autocleavage site 1 and SCC1 are shown as stick representation. **D,** Close-up view (red box in **B**) showing the autocleavage site 3 and SCC1 exosites binding to the TPR-like domain. Recognition of pS449 by the putative P-site 4 has been modelled in COOT. **E**, Cleavage assay of SCC1 mutant with alanine mutations in the LPE motif. **F,** Cleavage assay of SCC1 under varying salt concentrations. KCl concentrations ranged from 15 mM to 195 mM, demonstrating the effect of ionic strength on SCC1 cleavage efficiency. **G,** Affinity measurement of unphosphorylated and phosphorylated SCC1$^{100\text{-}320}$ binding to Separase^{C2029S} using fluorescence polarization. Each experiment was repeated three times; data points indicate mean +/- SEM.

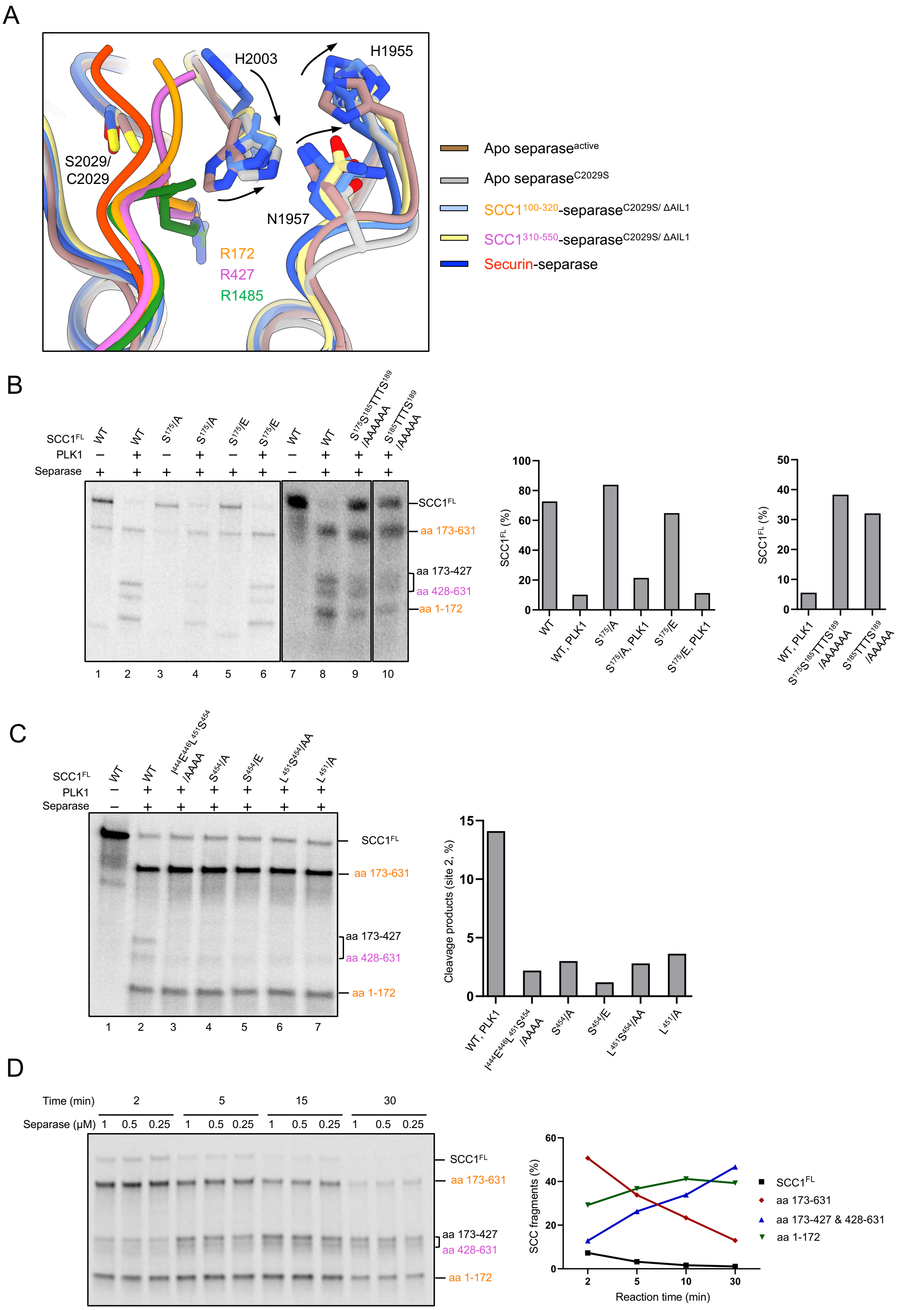

Figure S10. Cleavage assay of SCC1 mutants disrupting the interaction with separase. A, Residue arrangement around the catalytic site. The protease domains of apo-separase (active and inactive),

securin-separase and SCC1-separase complexes are superimposed and residues around the catalytic site are shown as sticks. Black arrows indicate the movement of H2003, H1955 and N2958 upon substrate binding. **B,** Cleavage assay of SCC1 mutants (phosphorylation sites) near cleavage site 1. Left, autoradiograph of SCC1 cleavage. Right, quantification of uncleaved SCC1. **C,** Cleavage assay of SCC1 mutants (phosphorylation sites) near cleavage site 2. Left, autoradiograph of SCC1 cleavage. Right, quantification of low molecular cleavage products of SCC1. **D,** Cleavage assay of wild-type SCC1 at different concentrations and time points. Left, autoradiograph of SCC1 cleavage. Right, quantification analysis of low molecular weight fragments from different time points at a concentration of 0.5 μM separase.

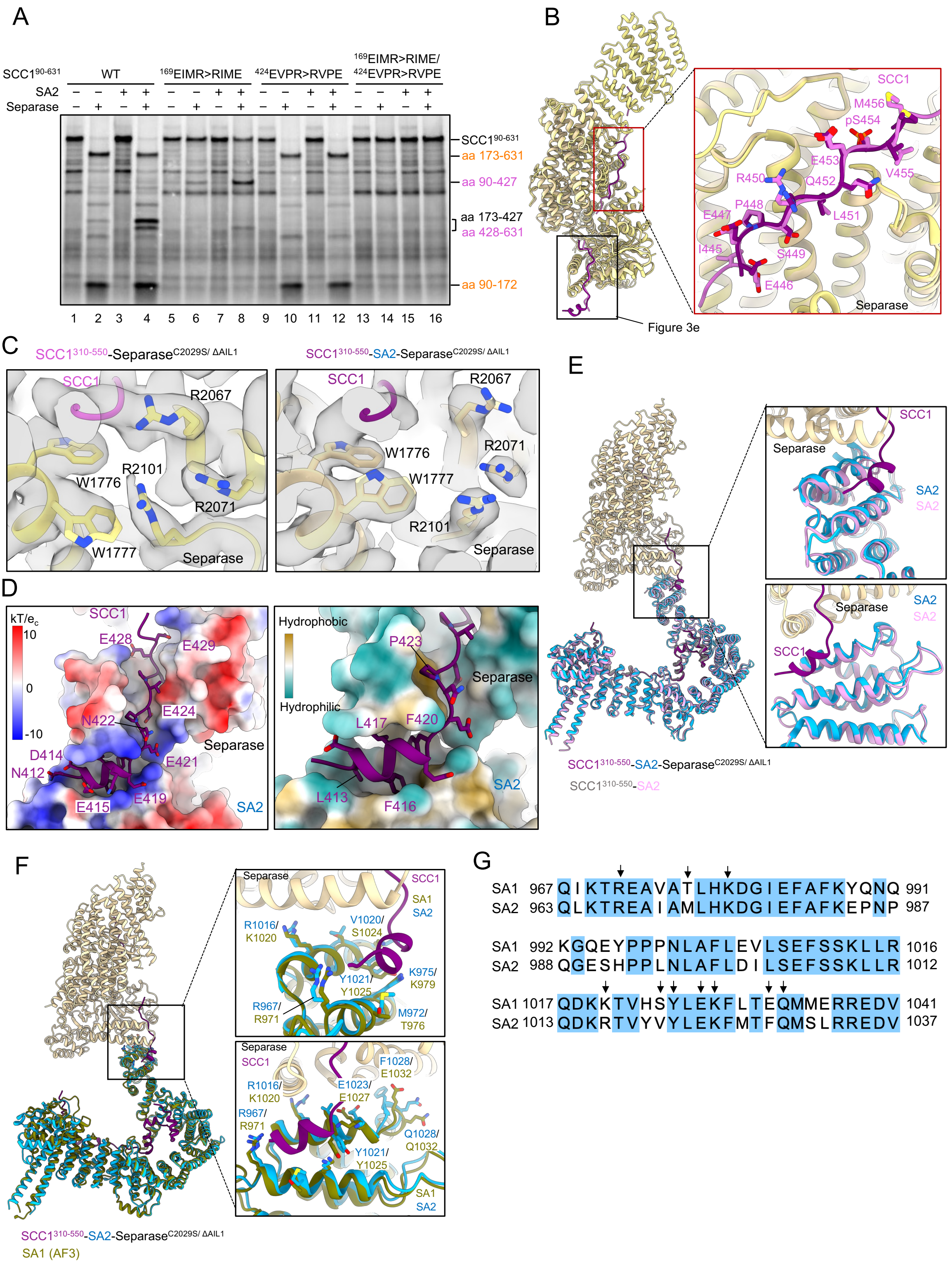

Figure S11. SA2 stimulates the cleavage of SCC1 at site 2. A, Cleavage assay of ^{35}S-labelled wild-type SCC1 (90-631 aa) and mutants. SCC1 was not phosphorylated but SA2 protein was added in the reactions as indicated. SCC1 mutants for cleavage sites 1 and 2 were generated by swapping the negatively charged glutamate and positively charged arginine in the conserved ExxR motif. **B,** Structural comparison between SCC1$^{310\text{-}550}$-SA2-separase$^{C2029S/\Delta AIL1}$ and SCC1$^{310\text{-}550}$-separase$^{C2029S/\Delta AIL1}$

complexes. The two structures were aligned using separase as a reference and are shown as ribbon representation. Black box, close-up view showing SCC1 binding to the protease domain, shown in **Fig. 3E**. Red box, close-up view showing SCC1 binding to the TPR-like domain of separase. SCC1 is depicted as sticks. **C,** Rotamer conformation changes of separase residues interacting with SCC1. Left, the rotamer conformations of W1777, R2067 and R2101 in the SCC1$^{310\text{-}550}$-separase$^{C2029S/\Delta AIL1}$ complex. Right, the rotamer conformations of W1777, R2067 and R2101 in the SCC1$^{310\text{-}550}$-SA2-separase$^{C2029S/\Delta AIL1}$ complex. The corresponding EM densities are shown in grey. **D**, Electrostatic surface potential and the hydrophobicity of the interaction interface. Separase and SA2 are shown as electrostatic surface or hydrophobic surface representations, while SCC1 is shown as cartoon. Electrostatic potentials are contoured from -10 (red) to +10 kTe-1 (blue). Hydrophobic surface is coloured from cyan (hydrophilic) to brown (hydrophobic). **E,** SA2 retains similar conformations before and after binding to separase. The SCC$^{310\text{-}550}$-SA2 complex was aligned to the tertiary complex using SA2 as reference. Black boxes, close-up views of the interaction interfaces. **F,** Residues of SA2 interacting with SCC1 and separase are conserved in SA1. The structure of SA1 (olive) predicted by Alphafold 3 was aligned to the tertiary complex using SA2 as reference. Flexible regions of SA1 were omitted for clarity. Black boxes, close-up views of interaction interfaces between SA2, SCC1 and separase with critical residues shown as sticks. **G,** Sequence alignment of SA1 and SA2 at C-terminal regions. Conservation between the sequences is highlighted in blue, with residues mediating interactions with separase indicated by black arrows.

395

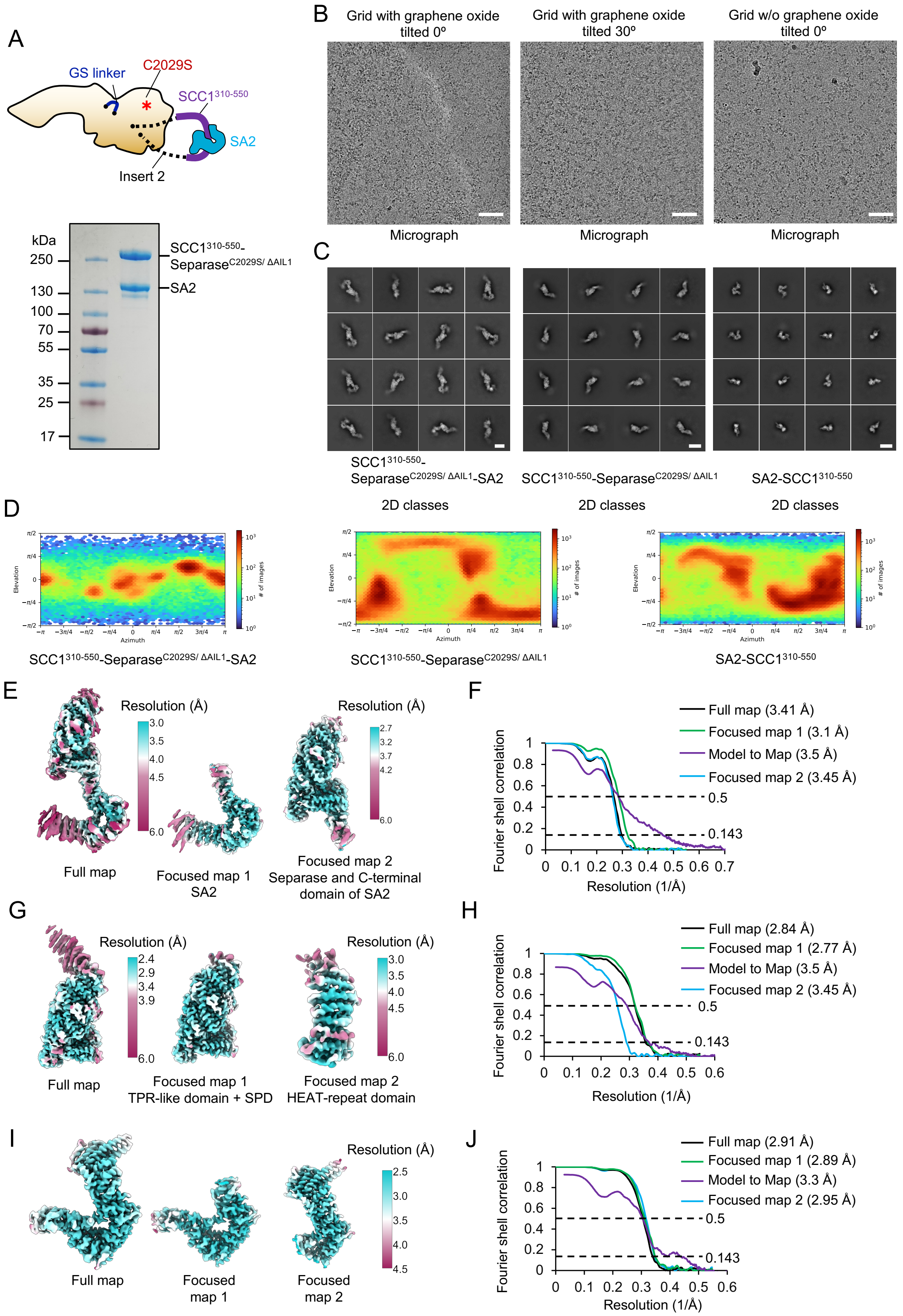

A
GS linker
C2029S
SCC1 310-550
SA2
Insert 2
kDa
250
130
100
70
55
35
25
17
SCC1 310-550-Separase C2029S/ ΔAIL1
SA2
B
Grid with graphene oxide tilted 0º
Grid with graphene oxide tilted 30º
Grid w/o graphene oxide tilted 0º
Micrograph
Micrograph
Micrograph
C
SCC1 310-550-Separase C2029S/ ΔAIL1-SA2
SCC1 310-550-Separase C2029S/ ΔAIL1
SA2-SCC1 310-550
2D classes
2D classes
2D classes
D
SCC1 310-550-Separase C2029S/ ΔAIL1-SA2
SCC1 310-550-Separase C2029S/ ΔAIL1
SA2-SCC1 310-550
E
Resolution (Å)
Full map
Focused map 1 SA2
Focused map 2 Separase and C-terminal domain of SA2
F
Full map (3.41 Å)
Focused map 1 (3.1 Å)
Model to Map (3.5 Å)
Focused map 2 (3.45 Å)
Fourier shell correlation
Resolution (1/Å)
G
Resolution (Å)
Full map
Focused map 1 TPR-like domain + SPD
Focused map 2 HEAT-repeat domain
H
Full map (2.84 Å)
Focused map 1 (2.77 Å)
Model to Map (3.5 Å)
Focused map 2 (3.45 Å)
Fourier shell correlation
Resolution (1/Å)
I
Resolution (Å)
Full map
Focused map 1
Focused map 2
J
Full map (2.91 Å)
Focused map 1 (2.89 Å)
Model to Map (3.3 Å)
Focused map 2 (2.95 Å)
Fourier shell correlation
Resolution (1/Å)

Figure S12. Biochemical and cryoEM analysis of SCC1$^{310-550}$-SA2 complex bound to separase. A, Schematic representation and SDS-PAGE gel of the SCC1$^{310-550}$-SA2-separase$^{C2029S/\Delta AIL1}$ complex. **B,** Representative cryo-electron micrographs collected on graphene oxide-coated EM grids with the stage untilted and tilted at 30°, and uncoated EM grids without stage tilting. Scale bars, 500 Å. **C,** Gallery of two-dimensional class averages of the tertiary complex (left), SCC1$^{310-550}$-separase$^{C2029S/\Delta AIL1}$ complex (middle) and SCC1$^{310-550}$-SA2 complex (right). Scale bars, 100 Å. **D,** Angular distribution plots for the tertiary complex (left), SCC1$^{310-550}$-separase$^{C2029S/\Delta AIL1}$ complex (middle) and SCC1$^{310-550}$-SA2 complex (right) calculated using non-uniform refinement algorithm in CryoSPARC (*47*). **E-J,** EM density maps of the three complexes colour-coded according to local resolution (**E, G, I**) and gold standard FSC curves for the full map and the focussed refined maps (**F, H, J**). The FSC curves between the full cryoEM map and the final atomic coordinates were calculated using Mtriage (*58*).

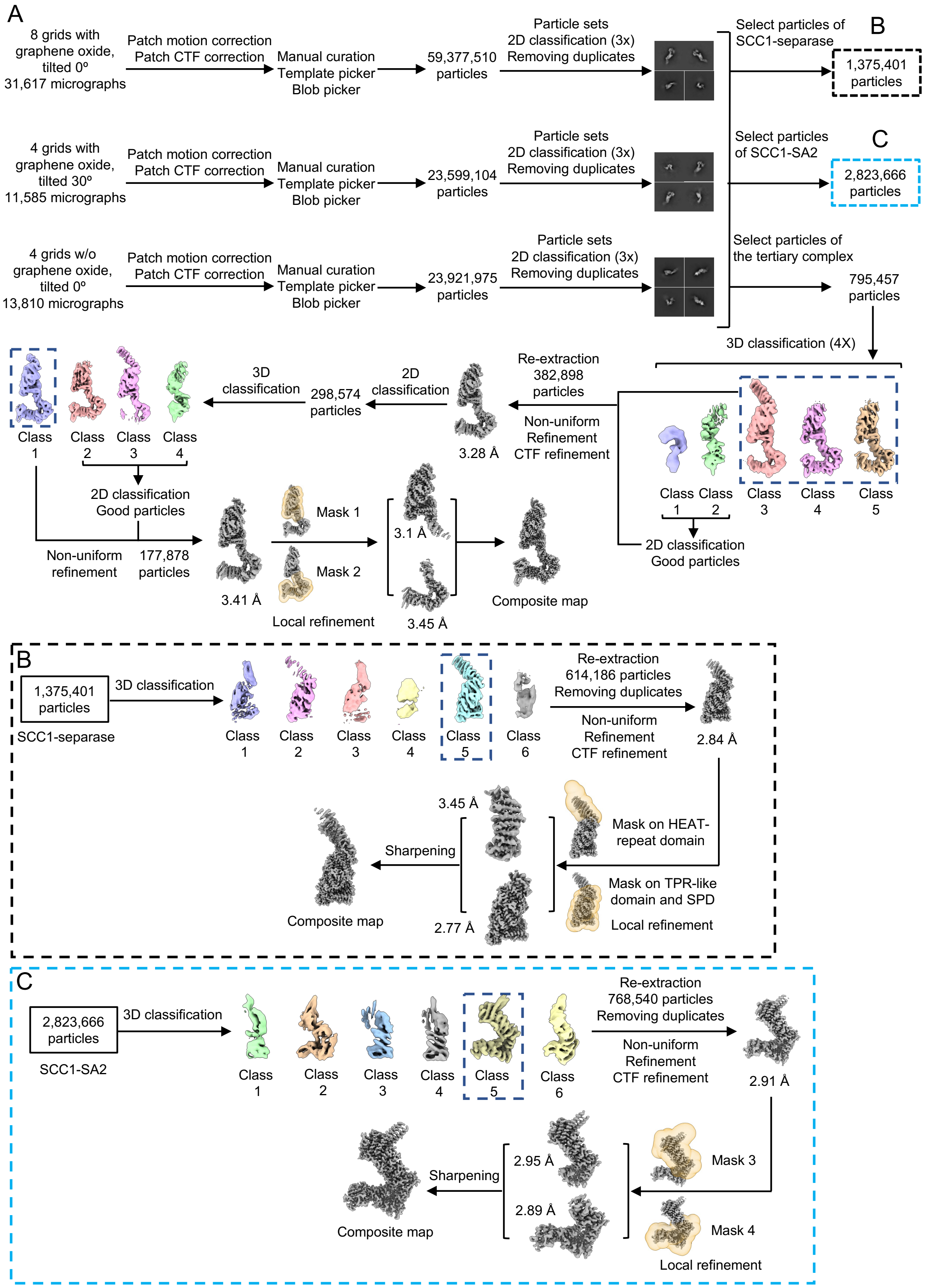

Figure S13. Data-processing flowcharts for SCC1$^{310-550}$-SA2 complex bound to separase. A, CryoEM processing pipeline of SCC1$^{310-550}$-SA2-separase$^{C2029S/\Delta AIL1}$ complex. Untilted and tilted data sets were initially processed separately. After 2D classification, particles belonging to the tertiary

complex, SCC1-separase and SCC1-SA2 complexes were separated from each other. Particles from each class were selected and combined from all data sets for further processing. Processing of SCC1-separase and SCC1-SA2 complex particles is detailed in panels **B** (black dashed box) and **C** (cyan dashed box), respectively. A total of 795,457 particles of the tertiary complex were subjected to five rounds of 3D classification followed by non-uniform refinement and CTF refinement, producing a map at 3.4 Å resolution. Local refinement using a mask covering SCC1, separase and C-terminal helices of SA2 further improved the density at the interaction interface. A composite map was created by combining two focussed refined maps. **B,** CryoEM processing pipeline of $SCC1^{310-550}$-$separase^{C2029S/\Delta AIL1}$ complex resulting in a map of 2.8 Å resolution. Masked refinements on the HEAT-repeat domain and the C-terminal TPR-like and protease domain was used to further improve the EM density. **C,** CryoEM processing pipeline of $SCC1^{310-550}$-SA2 complex. Non-uniform refinement yielded a map a 2.9 Å resolution. Focussed masks are shown on top of the 3D volumes.

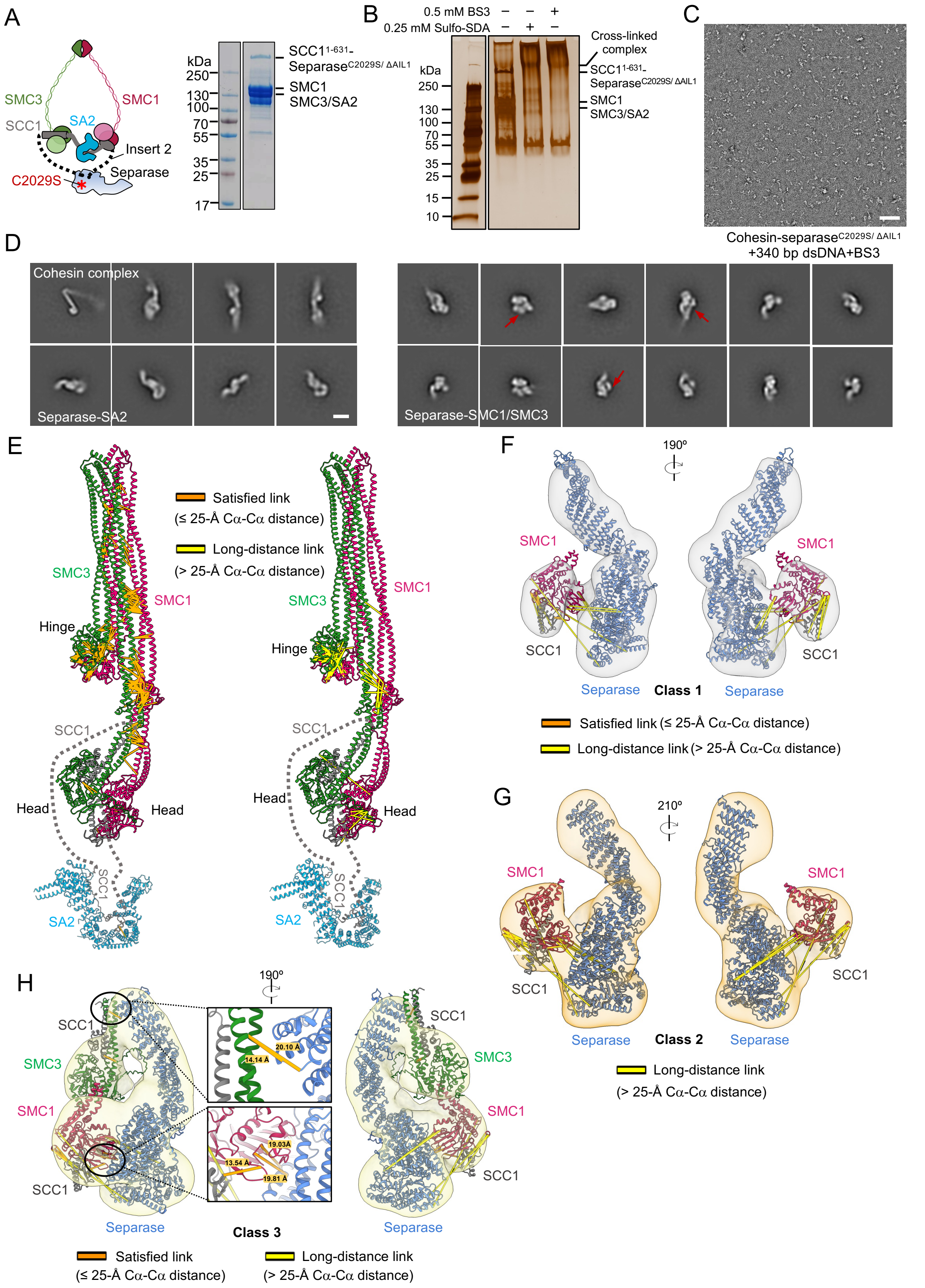

461
462 **Figure S14. Biochemical and XL-MS analysis of the cohesin-separase complex. A,** Schematic
463 representation and SDS-PAGE gel of the cohesin-separase complex. **B,** Silver stained SDS-PAGE gel

of cohesin-separase complex cross linked with 0.25 mM sulfo-SDA and 0.5 mM BS3. **C,** Representative negative staining micrograph of cohesin-separase complex incubated with 340 bp double-stranded DNA and 1 mM BS3. Scale bars, 100 nm. **D,** Two-dimensional class averages of the cohesin complex and separase bound to SA2 and potentially SMC subunits. Scale bars, 100 Å. Red arrows indicate the additional densities binding to separase. **E**, Mapping of sulfo-SDA cross-links with Cα-Cα distances ≤ 25 Å (orange lines) and > 25 Å (yellow lines) on the predicted structure of the cohesin complex. The flexible region of SCC1 was omitted and indicated by dashed lines. **F-H**, Three classes of EM maps resulting from negative staining analysis and cross-links mapped on corresponding models. AF3-predicted models of full-length human separase and the head domains of SMC1/3 in complex with SCC1 were fitted into the density maps and shown as cartoon. Satisfied Sulfo-SDA cross-links with Cα-Cα distances ≤ 25 Å are shown as orange solid lines, whereas long-distance cross-links are shown as solid yellow lines.

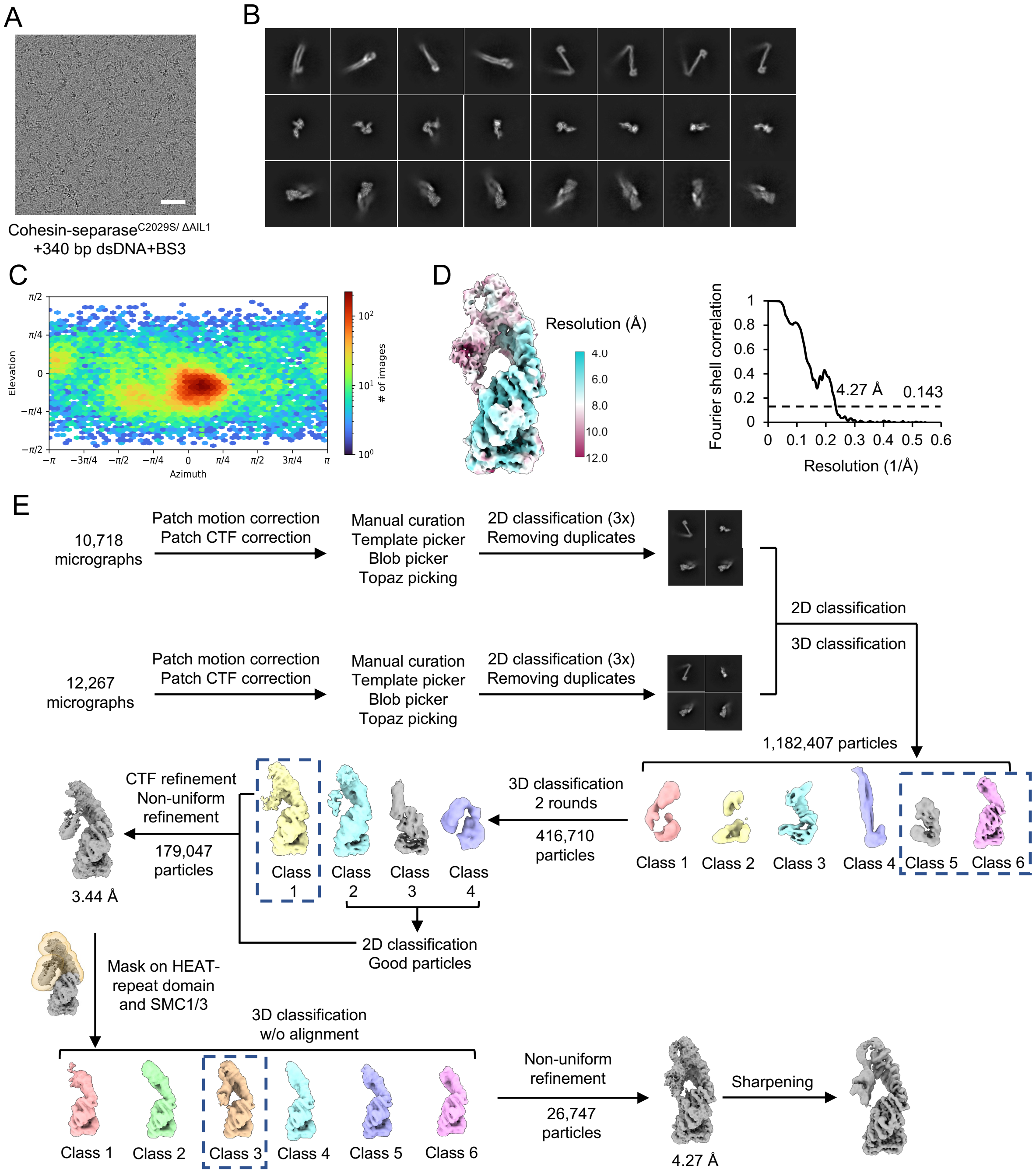

Figure S15. CryoEM analysis of the cohesin-separase complex. A, Representative EM micrograph of the cohesin-separase complex incubated with 340 bp double-stranded DNA and cross-linked 1 mM BS3. Scale bars, 500 Å. **B,** Representative two-dimensional class averages of the cohesin complex and separase bound to SMC3. Scale bars, 100 Å. **C,** Angular distribution plots for cohesin-separase complex data set calculated using non-uniform refinement algorithm in CryoSPARC (*47*). **D,** EM density maps of cohesin-separase complex colour-coded according to local resolution (right) and gold standard FSC curves for the full map (left). **E,** CryoEM processing pipeline of cohesin-separase complex. To improve the density of SMC3 bound to separase, 3D classification without alignment was performed with a mask covering SMC3 and the HEAT-repeat domain of separase.

Table S1. CryoEM data collection, refinement, and validation statistics

	Apo separase^{active}	Apo separase^{C2029S}	SCC1$^{100-320}$-separase$^{C2029S/\Delta AIL1}$ (phosphorylated)	SCC1$^{310-550}$-separase$^{C2029S/\Delta AIL1}$ (phosphorylated)	Cohesin-separase$^{C2029S/\Delta AIL1}$
**Data collection**					
Microscope	Talos Arctica	Talos Arctica	Talos Arctica	Talos Arctica	Talos Arctica
Magnification	150,000x	150,000x	130,000x	130,000x	130,000x
Voltage (keV)	200	200	200	200	200
Electron dose ($e^-/Å^{-2}$)	40	40	50	50	50
Detector	Falcon 3	Falcon 3	Falcon 4i	Falcon 4i	Falcon 4i
Energy filter	–	–	Selectris X	Selectris X	Selectris X
Pixel size (Å/pixel)	0.9759	0.9759	0.9024	0.9024	0.9024
Defocus range (μm)	0.6-2.2	0.6-2.2	0.6-2.0	0.6-2.0	0.8-2.4
Number of micrographs	3,314	5,292	12,867	7,451	22,985
**Reconstruction**					
Particles	224,027	118,288	195,231	298,574	26,747
Box size (pix)	360	360	400	400	512
Resolution (global, Å)	3.3	3.1	3.0	2.9	4.3
Resolution range	3.0-6.0	2.9-6.0	2.8-6.0	2.8-7.0	4.0-10.0
(local, Å)	0.143	0.143	0.143	0.143	0.143
FSC threshold					
**Model composition**					
Protein residues	1400	1340	1518	1459	
**Refinement**					
Resolution (Å)	4.0	3.6	3.5	3.6	
FSC threshold	0.5	0.5	0.5	0.5	
Model to map scores					
-CC	0.77	0.78	0.74	0.71	
B factors ($Å^2$)					
Protein residues	-85.48	-87.57	-85.82	-86.44	
Ligand	-61.83	-61.83	-61.83	-61.83	
R.m.s deviations					
Bond lengths (Å)	0.005	0.005	0.007	0.007	
Bong angles (°)	0.998	0.694	0.848	0.893	
**Validation**					
Clashscore, all atoms	7.0	4.0	7.0	8.0	
Rotamer outliers (%)	0.5	0.5	0.3	0.6	
**Ramachandran plot**					
Favoured (%)	96.03	97.28	97.86	96.73	
Allowed (%)	3.97	2.64	2.14	3.27	
Outliers (%)	0.00	0.08	0.00	0.00	
**Deposition**					
PDB ID	9HMA	9HM7	9HN5	9HN4	
EMDB ID	EMD-52290	EMD-52288	EMD-52307	EMD-52306	

SCC1$^{310-550}$-SA2-separase$^{C2029S/\Delta AIL1}$

**Data collection**									
Microscope	Talos Arctica			Talos Arctica			Talos Arctica		
Magnification	130,000x			130,000x			130,000x		
Voltage (keV)	200			200			200		
Electron dose (e$^-$/Å^{-2})	50			50			50		
Detector	Falcon 4i			Falcon 4i			Falcon 4i		
Energy filter	Selectris X			Selectris X			Selectris X		
Pixel size (Å/pixel)	0.9024			0.9024			0.9024		
Defocus range (μm)	0.6-2.0			1.0-2.6			0.6-2.0		
Support layer	Graphene oxide			Graphene oxide			w/o graphene oxide		
Stage tilted (°)	0			30			0		
Number of micrographs	31,617			11,585			13,810		
**Reconstruction**									
Complex	SCC1$^{310-550}$-SA2-separase$^{C2029S/\Delta AIL1}$				SCC1$^{310-550}$-separase$^{C2029S/\Delta AIL1}$				SCC1$^{310-550}$-SA2
Maps	Composite map	Consensus map	Focus-refined map 1	Focus-refined map 2	Composite map	Consensus map	Focus-refined map 1	Focus-refined map 2	
Particles	177,878	177,878	177,878	177,878	614,186	614,186	614,186	614,186	768,540
Box size (pix)	512	512	512	512	400	400	400	400	400
Resolution (global, Å)	-	3.4	3.1	3.5	-	2.8	2.8	3.5	2.9
Resolution range (local, Å)	-	2.9-11.5	2.8-18.3	3.0-20.0	-	2.4-10.0	2.4-15.0	2.9-16.9	2.5-9.0
FSC threshold	-	0.143	0.143	0.143	-	0.143	0.143	0.143	0.143
**Model composition**									
Protein residues	2181				1692				1013
**Refinement**									
Resolution (Å)	3.5				3.5				3.3
FSC threshold	0.5				0.5				0.5
Model to map scores -CC	0.75				0.76				0.80
B factors (Å^2)									
Protein residues	-82.10				-87.94				-72.90
Ligand	-61.83				-61.83				--
R.m.s deviations									
Bond lengths (Å)	0.007				0.007				0.013
Bong angles (°)	1.019				0.895				1.526
**Validation**									
Clashscore, all atoms	12.0				7.0				7.0
Rotamer outliers (%)	0.7				0.4				0.7
**Ramachandran plot**									
Favoured (%)	93.87				97.08				96.61
Allowed (%)	6.13				2.92				3.19
Outliers (%)	0.00				0.00				0.20
**Deposition**									
PDB ID	9HMS				9HN0				9HMV
EMDB ID	EMD-52297	EMD-52295	EMD-52291	EMD-52294	EMD-52303	EMD-52302	EMD-52300	EMD-52301	EMD-52298

	SCC1$^{1-631}$-separase$^{C2029S/\Delta autocleavage}$ fusion
**Data collection**	
Microscope	Talos Arctica
Magnification	150,000x
Voltage (keV)	200
Electron dose ($e^-/Å^{-2}$)	40
Detector	Falcon 3
Energy filter	–
Pixel size (Å/pixel)	0.9759
Defocus range (μm)	0.6-2.2
Number of micrographs	5,876
**Reconstruction**	
Particles	131,299
Box size (pix)	360
Resolution (global, Å)	3.5
Resolution range	3.3-8.0
(local, Å)	0.143
FSC threshold	
**Model composition**	
Protein residues	1396
**Refinement**	
Resolution (Å)	4.1
FSC threshold	0.5
Model to map scores	
-CC	0.75
B factors ($Å^2$)	
Protein residues	-85.42
Ligand	-61.83
R.m.s deviations	
Bond lengths (Å)	0.004
Bong angles (°)	0.686
**Validation**	
Clashscore, all atoms	8.0
Rotamer outliers (%)	0.7
**Ramachandran plot**	
Favoured (%)	96.16
Allowed (%)	3.84
Outliers (%)	0.00
**Deposition**	
PDB ID	9HVY
EMDB ID	EMD-52445

Table S2. Structures of apo-separase and separase bound to inhibitors or substrates

**Protein complexes**	**RMSD Cα atoms (vs apo separase^{active})**	**Resolution (Å)**	**PDB ID**
Apo separase^{active}	–	3.3	9HMA
Apo separase^{C2029S}	0.739	3.1	9HM7
Securin-separase	1.062	2.9	7NJ1
CCC-securin$^{\Delta 160}$-separase^{C2029S}	0.942	3.6	7NJ0
SCC1$^{100\text{-}320}$-separase$^{C2029S/\Delta AIL1}$ (phosphorylated)	0.994	3.0	9HN5
SCC1$^{310\text{-}550}$-separase$^{C2029S/\Delta AIL1}$ (phosphorylated)	1.021	2.9	9HN4
SCC1$^{310\text{-}550}$-separase$^{C2029S/\Delta AIL1}$	0.905	2.8	9HN0
SCC1$^{310\text{-}550}$-SA2-separase$^{C2029S/\Delta AIL1}$	0.813	3.4	9HMS

Table S3. Summary of key residues in securin, SCC1, the autoinhibitory loop 2 (AIL2) and autocleavage sites of separase binding to P-sites 1-5.

	P-site 1	P-site 2	P-site 3	P-site 4	P-site 5
**SCC1 site 1**	D166, E169	(p)S175	(p)S185	(p)S189	E194
**SCC1 site 2**	E421, E424	D429	E446	(p)S449	(p)S454
**Autocleavage sites 1 and 3**	E1483	-	E1530	E1532	D1537
**Securin**	E113, E115	-	E133	-	-
**AIL2**	-	-	-	-	D972

(p): Phosphorylation of S175, S185, S189, S449 and S454 in SCC1 detected by mass spectrometry analysis or observed in cryoEM structures.

Data S1. (separate file)
Crosslinking mass spectrometry raw data. A spreadsheet containing all measured protein-protein crosslink pairs.

Movie S1. (separate file)
Movie illustrating the different binding modes of separase binding partners to separase. The movie shows a morph between binding of securin, SCC1 site 1, SCC1 site 2, and separase autocleavage sites to separase. Specific substrate-docking sites on separase such as the P-sites 1-5 are highlighted for SCC1 site 1 and site 2.

Movie S2. (separate file)
Movie illustrating the binding interface between separase, SCC1 and SA proteins. The movie provides a structural rational why SCC1 site 2 cleavage by separase is stimulated in the presence of SA2. SA2 provides extra binding sites and thereby locks SCC1 in a position susceptible for cleavage.
